# Comparison of RNA-Seq and Microarray Gene Expression Platforms for the Toxicogenomic Evaluation of Liver From Short-Term Rat Toxicity Studies

**DOI:** 10.3389/fgene.2018.00636

**Published:** 2019-01-22

**Authors:** Mohan S. Rao, Terry R. Van Vleet, Rita Ciurlionis, Wayne R. Buck, Scott W. Mittelstadt, Eric A. G. Blomme, Michael J. Liguori

**Affiliations:** Investigative Toxicology and Pathology, Global Preclinical Safety, AbbVie, North Chicago, IL, United States

**Keywords:** RNA-Seq, microarray, toxicogenomics, liver toxicity, DEG, non-coding transcripts, IPA, *in vivo*

## Abstract

Gene expression profiling is a useful tool to predict and interrogate mechanisms of toxicity. RNA-Seq technology has emerged as an attractive alternative to traditional microarray platforms for conducting transcriptional profiling. The objective of this work was to compare both transcriptomic platforms to determine whether RNA-Seq offered significant advantages over microarrays for toxicogenomic studies. RNA samples from the livers of rats treated for 5 days with five tool hepatotoxicants (α-naphthylisothiocyanate/ANIT, carbon tetrachloride/CCl_4_, methylenedianiline/MDA, acetaminophen/APAP, and diclofenac/DCLF) were analyzed with both gene expression platforms (RNA-Seq and microarray). Data were compared to determine any potential added scientific (i.e., better biological or toxicological insight) value offered by RNA-Seq compared to microarrays. RNA-Seq identified more differentially expressed protein-coding genes and provided a wider quantitative range of expression level changes when compared to microarrays. Both platforms identified a larger number of differentially expressed genes (DEGs) in livers of rats treated with ANIT, MDA, and CCl_4_ compared to APAP and DCLF, in agreement with the severity of histopathological findings. Approximately 78% of DEGs identified with microarrays overlapped with RNA-Seq data, with a Spearman’s correlation of 0.7 to 0.83. Consistent with the mechanisms of toxicity of ANIT, APAP, MDA and CCl_4_, both platforms identified dysregulation of liver relevant pathways such as Nrf2, cholesterol biosynthesis, eiF2, hepatic cholestasis, glutathione and LPS/IL-1 mediated RXR inhibition. RNA-Seq data showed additional DEGs that not only significantly enriched these pathways, but also suggested modulation of additional liver relevant pathways. In addition, RNA-Seq enabled the identification of non-coding DEGs that offer a potential for improved mechanistic clarity. Overall, these results indicate that RNA-Seq is an acceptable alternative platform to microarrays for rat toxicogenomic studies with several advantages. Because of its wider dynamic range as well as its ability to identify a larger number of DEGs, RNA-Seq may generate more insight into mechanisms of toxicity. However, more extensive reference data will be necessary to fully leverage these additional RNA-Seq data, especially for non-coding sequences.

## Introduction

Toxicogenomics has been used as a tool in pre-clinical toxicity assessment for almost two decades (Kolaja and Kramer, 2002; Suter et al., 2004; Yang et al., 2004). The objective of toxicogenomics in pharmaceutical research and development (R&D) is to identify differentially expressed genes (DEGs) in tissues or cells exposed to test agents in order to predict toxic effects, to generate a mechanistic understanding of toxic effects, or to identify biomarkers of toxicity (Buck et al., 2008). Traditionally, hybridization-based approaches such as microarrays have been the standard gene expression profiling technology for use in toxicogenomics (Nuwaysir et al., 1999; Yang et al., 2004; Liguori et al., 2005; Waring et al., 2006). More recently, RNA-Seq has emerged as an alternative method for gene expression profiling (Merrick et al., 2013). The main difference between RNA-Seq and microarrays is that the former allows for full sequencing of the whole transcriptome while the latter only profiles predefined transcripts/genes through hybridization. This implies that RNA-Seq can help identify more differentially modulated transcripts of toxicological relevance, splice variants, and non-coding transcripts [e.g., microRNA (miRNA), long non-coding RNA (lncRNA), pseudogenes] and that these additional data may be informative for toxicity prediction, mechanistic investigations or biomarker discovery (Wang et al., 2010; Iyer et al., 2015; Li et al., 2015; Yan et al., 2015). Due to these advantages and general advancement of the field, there has been an increasing interest in using RNA-Seq platforms for toxicogenomic studies (Chen et al., 2012; Bisgin et al., 2018).

Several studies have compared DEGs identified from RNA-Seq and microarray platforms (Xu et al., 2013; Wang et al., 2014; Zhao et al., 2014; Hung and Weng, 2017). Likewise, there have been considerable advances in sample handing, data capture, and analytics of RNA-Seq data (Li and Dewey, 2011; Ritchie et al., 2015; Bray et al., 2016; Conesa et al., 2016; Ghosh and Chan, 2016). However, the RNA-Seq approach has a few disadvantages compared to microarrays, namely (1) a lack of optimized and standardized protocols for analysis in spite of the availability of multiple computational tools (Chandramohan et al., 2013; Hayer et al., 2015), and (2) the size of RNA-Seq files, which are considerably larger than microarray files. Finally, RNA-Seq entails an extensive and more complex bioinformatic analysis, which results in highly intensive and expensive computation infrastructure and analytics, as well as longer analysis times (Robinson and Oshlack, 2010; Esteller, 2011; Garber et al., 2011; Trapnell et al., 2012). However, these limitations are gradually improving.

While RNA-Seq appears to be an attractive technology, it is unclear whether the technology results in substantial benefits compared to microarrays when used in toxicogenomic studies. Published studies have not systematically and thoroughly evaluated the concordance and discordance of DEGs between the two technologies in toxicological studies. Therefore, the objective of the current study was to compare the two technologies using RNA samples extracted from the liver of rats dosed with several prototypical hepatotoxicants selected for their distinct mechanisms of toxicity. Both technologies were assessed using a combination of bioinformatics tools and within the context of liver biology pathways.

## Materials and Methods

### Dose Selection

The DrugMatrix toxicogenomic database was used to select the doses for a-naphthylisothiocyanate (ANIT), carbon tetrachloride (CCl_4_), methylenedianiline (MDA), acetaminophen (APAP), and diclofenac (DCLF) (Ganter et al., 2005). This database holds comprehensive results from hundreds of highly controlled and standardized toxicological experiments in which rats or primary rat hepatocytes were treated with therapeutic, industrial, and environmental chemicals over multiple doses/time periods. Based upon the DrugMatrix database, in male rats, ANIT, APAP, MDA, CCl_4_, and DCLF alter the expression of genes involved in liver toxicity and/or increased serum transminases at 60, 1175, 81, 972, and 10 mg/kg, respectively. Thus, doses close to these values were selected for our *in vivo* experiments.

### In-Life Studies

All animal experiments for this study were conducted in accordance with the *Guide for the Care and Use of Laboratory Animals*. All studies were approved by AbbVie’s Institutional Animal Care and Use Committee (IACUC). Briefly, male Sprague Dawley rats were purchased from Charles River Laboratories, Inc. (Portage, MI, United States). The rats were typically 6 to 8 weeks of age and weighed 250–350 g at the start of the study. The animals were acclimated for a minimum of 2 days after receipt and randomized into treatment and control groups. Rats were permitted certified rodent chow and water *ad libitum* and were fasted overnight prior to necropsy.

Male rats (*n* = 3/group) were treated by oral gavage for 5 days with ANIT, 100 mg/kg in corn oil; APAP, 1000 mg/kg/day in corn oil; DCLF, 10 mg/kg/day in water; CCl_4_, 1582 mg/kg/day in corn oil; MDA, 100 mg/kg/day in 35% ethanol (EtOH) (v/v). Corn oil-, EtOH- (*n* = 3/group) or water- (*n* = 2/group) vehicle treated rats served as controls. Rats were anesthetized 24 h after the last dose with isoflurane and blood samples were collected through the posterior vena cava for clinical pathological examinations. Rats were then humanely sacrificed through exsanguinations and necropsied. Liver samples (left lateral lobe) were flash frozen at necropsy. A portion of the liver from each animal was also fixed in 10% neutral buffered formalin and subsequently embedded in paraffin blocks for sectioning. Slides for hematoxylin and eosin (H&E) staining were prepared according to the well-established methods and examined by a veterinary pathologist certified by the American College of Veterinary Pathologists (WRB). Histopathological analyses were performed on these livers to confirm the presence of toxicity (Supplementary Figures S1A–E). Serum clinical chemistry parameters were quantified using an Abbott Aeroset clinical chemistry analyzer (Abbott Laboratories, Abbott Park, IL, United States) and individual animal data are provided in Supplementary Table S1.

### RNA Sample Preparation

Total RNA from ∼50 mg of flash frozen liver for all treated (*n* = 15) and vehicle (*n* = 11) animals was isolated by Qiazol extraction with on-column DNase I treatment (Qiagen, Redwood City, CA, United States) and evaluated for quality (RIN scores ≥ 9) by BioAnalyzer (Agilent, Santa Clara, CA, United States). Aliquots of the same total RNA samples were used as input for each platform.

### RNA-Seq Data Generation

Seventy five ng total RNA/liver was used as input for RNA-Seq library construction using the TruSeq Stranded mRNA Library Prep Kit (Illumina, San Diego, CA, United States) on the Neo-Prep Library Prep System. The library preparation kit used was optimized for enriching coding mRNAs. Libraries were normalized, pooled and loaded on a NextSeq500 for single read sequencing at 1 bp × 75 bp using a HI Output flowcell according to manufacturer’s protocols (Illumina, San Diego, CA, United States). FastQ files were generated and uploaded to Array Studio (OmicSoft, Cary, NC, United States) for analysis. On average 25 to 26 million NGS short reads were generated per sample (Supplementary Table S1).

The efficiency and accuracy of the alignment of NGS RNA-Seq short reads to a reference genome is an important component in any DEGs comparison studies. Computational alignment methods for RNA-Seq data analysis are constantly improving. OSA4 (Omicsoft, Cary, NC, United States) was used for alignment of RNA-Seq short reads to the rat reference genome (Hu et al., 2012).

The percentage of total reads uniquely mapped to the reference genome using OSA4 (Omicsoft, Cary, NC, United States) was between 86 and 91% for all 26 samples, supporting the good quality for the RNA-Seq reads. About 5.5 to 7.5% of the samples non-uniquely mapped and only 3 to 4% did not map (Supplementary Table S1). The NGS short reads mapped well onto 32,663 Ensembl.R83 annotated genes of the rat reference genome (Rat B6.0); of these, 22,271 were protein-coding sequences and the remaining 10,392 were non-coding transcripts. After removing redundant genes from the microarray probes, only approximately 18,700 annotated protein-coding genes overlapped between the two technologies. For platform comparison purposes, the gene symbol was used for this protein-coding set.

Maximum likelihood (ML) expression levels were estimated using an Expectation–Maximization (EM) algorithm integrated within the Omicsoft suite of programs (Hu et al., 2012). Generally, RNA-Seq reads do not span entire transcripts, and the transcripts from which they are derived are not always uniquely determined. Paralogous gene families, low-complexity sequences and high sequence similarity between alternatively spliced isoforms of the same gene are primary factors contributing to mapping uncertainty. Due to these factors, a significant number of reads are multi-reads. Two strategies have been previously used for handling gene multi-reads. First, simply discarding them, keeping only uniquely mapped reads for expression estimation. Second, rescuing multi-reads by allocating fractions of them to genes in proportion to coverage by uniquely mapping reads. The rescue strategies have been shown to give expression estimates that are in better agreement with microarrays than those from only using uniquely mapping reads (Hu et al., 2012) Consequently, we used an Omicsoft implemented rescue strategy for gene quantification. Raw Illumina RNA-Seq FASTQ files are available in the Gene Expression Omnibus (GEO) database (Edgar et al., 2002) with an Accession No. GSE122315.

### Microarray Data Generation

Microarray hybridization was performed using the standard protocol provided by Affymetrix, Inc. (Santa Clara, CA, United States). Briefly, 5 μg of total RNA from each rat liver was reverse transcribed into cDNA using a Superscript II Double-Strand cDNA synthesis kit (Invitrogen Life Technologies, Carlsbad, CA, United States) according to the manufacturer’s instructions, except that the primer used for the reverse transcription reaction was a modified T7 primer with 24 thymidines at the 5′ end (Affymetrix). The sequence was 5′-GGCCAGTGAATTGTAATACGAC-TCACTATAGGGAGGCGG-(dT)_24_-3′. cDNA was purified via MinElute filtration (Qiagen, Redwood City, CA, United States) and then used to synthesize biotin-labeled cRNA using the Enzo RNA Transcript Labeling Kit (Enzo Life Sciences, Farmingdale, NY, United States) according to the manufacturer’s instructions. Labeled cRNA was purified using RNeasy kits (Qiagen, Redwood City, CA, United States), and the cRNA concentration was evaluated. Labeled cRNA (20 μg) was then fragmented in a solution of 40 mM Tris-acetate, pH 8.1, 100 mM KOAc, and 30 mM MgOAc at 94°C for 35 min, and then hybridized to an Affymetrix rat genome RAE230 2.0 array, which contains sequences to approximately 31,000 probe sets, at 45°C overnight using an Affymetrix Hybridization Oven 640. We have consistently used this Affymetrix chip platform for toxicology studies for ease of cross-study comparison (Liguori et al., 2016), which is consistent with others in the pharmaceutical industry including much of the Drug Matrix database (Ganter et al., 2005; Sutherland et al., 2017). Arrays were subsequently washed, and stained with strepavidin-phycoerythrin (Molecular Probes, Carlsbad, CA, United States) using the GeneChip^®^ Fluidics Workstation 450 (Affymetrix), and finally scanned using the Affymetrix GeneChip^®^ Scanner 3000. CEL files were generated and uploaded to ArrayStudio (Omicsoft, Cary, NC, United States) for gene expression analysis. The microarray probes were annotated with both Refseq and Ensembl.R83 (Omicsoft, Cary, NC, United States). Raw microarray CEL data files are also available in the GEO database with an accession number .

### Principal Component Analysis (PCA)

Principal component analysis (PCA) calculations were performed to assess the quality of the RNA-Seq and microarray data using Array Studio (Omicsoft, Cary, NC, United States) and SAS Enterprise Guide 6.1 (SAS Institute, Cary, NC, United States). Using SAS Enterprise Guide 6.1, a two sample *t*-test was performed to compute a *p*-value for the major principal component of RNA-Seq and microarray datasets (toxicant treated samples vs. control). The computed *p*-values were 0.002 and 0.004 for the RNA-Seq and microarray datasets (toxicant treated samples vs. control), respectively. The principal components were visualized in the Spotfire (Tibco, Inc., Palo Alto, CA, United States) data analysis tool.

### Gene Expression Analysis

Differentially expressed genes for each platform were identified by comparison of the mean of expression intensities or counts of compound-treated samples to the mean of expression intensities or counts of the corresponding vehicle-treated samples with a fold change (FC) > 1.5 and *p* < 0.01. The resulting genes were analyzed for concordance and discordance using Array Studio 10.1, SAS Enterprise Guide 6.1, Spotfire (Tibco, Inc., Palo Alto, CA, United States) and JMP 10 (Cary, NC, United States). Further pathway and gene-enrichment analyses were conducted using Ingenuity Pathway Analysis (IPA) version 31813283 (Qiagen, Redwood City, CA, United States) and SAS EG. 6.1 (SAS Institute, Cary, NC, United States). In IPA, the significance of the association between the DEGs and the canonical pathway was computed using two parameters, namely: (1) a ratio of the number of DEGs from the data set that map to the pathway divided by the total number of genes that constitute the canonical pathway and (2) a −log_10_ (*p*-value) determining the probability that the association between the DEGs in the data set and the canonical pathway is due to chance alone. In the present analysis, the computed −log_10_ (*p*-value) of 3.0 (*p* < 0.001) and above was considered as statistically significant.

The Causal analysis (upstream analysis) of IPA examines how many known targets of each transcription regulator are present in the DEGs identified by RNA-Seq and microarrays, and also compares their direction of change (i.e., expression in the experimental sample(s) relative to control) to what is expected from the literature in order to predict likely relevant transcriptional regulators. IPA used z-score algorithm to make predictions (Krämer et al., 2013) – with z-scores greater than 3 (activated) or smaller than −3 (inhibited) being considered to be significant.

### Statistical Analyses

#### Microarray

All chips were normalized using the Robust Multi-array Average (RMA) method (Bolstad et al., 2003) implemented in Array Studio. All data processing was performed using Array Studio software. Mean expression levels were obtained by calculating the geometrical means of the RMA-normalized data for toxicant treated and control sample groups, respectively. A two-sided *t*-test was performed using the inference module of Array Studio, to determine which genes were significantly differentially expressed between the toxicant treated and control groups, and Benjamini–Hochberg false discovery rate (FDR) multiple testing correction and alpha level of 0.05 was applied.

#### RNA-Seq

Voom module implemented within Array Studio (Omicsoft, Cary, NC, United States) was used. This module transforms count data log 2 transformed counts per million (logCPM), robustly estimates the mean-variance relationship and generates a precision weight for each individual normalized observation. Inference tests based on the Voom algorithm were applied to adjust read depth differences between samples and estimate changes or differences of gene expression when comparing sample groups. Genes with little or no expression [average transcripts per million (TPM) < 0.1] were excluded from inference tests. DEGs from the inference test were selected according to expression changes of more than 1.5 and Benjamini–Hochberg FDR multiple correction and alpha level of 0.05 was applied.

## Results

### Histopathology

Administration of ANIT, MDA, and CCl_4_ resulted in expected hepatotoxicity characterized by changes in relevant serum chemistries at necropsy. Increases (>2X) in serum activities of hepatocellular leakage enzymes [alanine aminotransferase (ALT), aspartate aminotransferase (AST), glutamate dehydrogenase (GLDH)] were observed with ANIT, CCl_4_, and MDA. Increases in total serum bilirubin were also observed for ANIT and MDA, and minimally for APAP. A complete listing of serum chemistry values is presented in Supplementary Table S2. These changes in serum chemistry values were consistent with the histopathologic findings which were detected in the livers from rats treated with ANIT, MDA, and CCl_4_, but not APAP and DCLF. Supplementary Figures S1A–E provides representative photomicrographs illustrating the histopathological appearance of the liver from a representative rat in each dose group. ANIT (Supplementary Figure S1A) and MDA (Supplementary Figure S1B) treatment resulted in biliary toxicity characterized by hypertrophy and hyperplasia of the bile duct epithelium, and infiltration of neutrophils into the pericholangiolar space and bile duct lumina. CCl_4_ (Supplementary Figure S1C) treatment resulted in widespread centrilobular hepatocellular macrovesicular and microvesicular steatosis. Neither APAP (Supplementary Figure S1D), nor DCLF treatment (Supplementary Figure S1E) resulted in histological findings, suggesting that higher dose levels would have been required for these hepatotoxicants to induce liver effects in male rats after five daily doses. The dose response of tolerated hepatotoxicity of DCLF is narrow; 40 mg/kg/day is poorly tolerated, with only one of three animals surviving after five daily doses (data not shown). The APAP and DCLF samples were nonetheless further analyzed since they were considered useful to better understand the sensitivity and utility of transcriptomic changes in rat toxicity studies.

### Principal Component Analysis of RNA-Seq and Microarray Data

**FIGURE 1:**
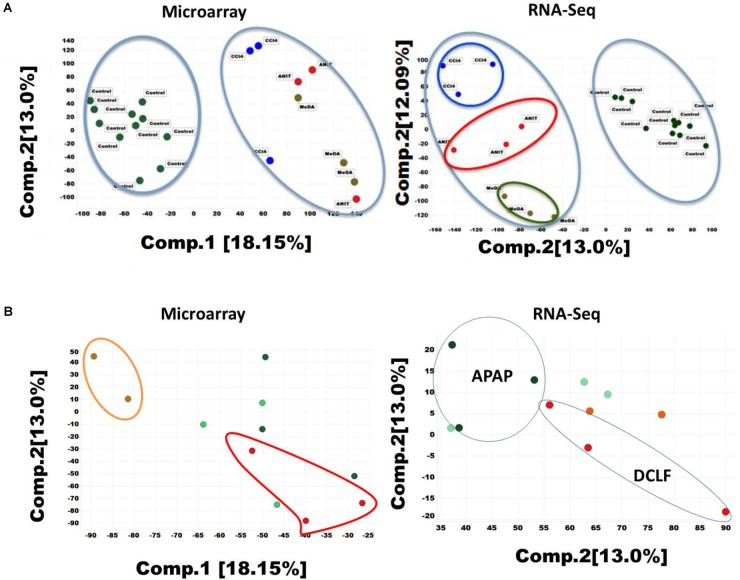
**(A)** Principal component analysis (PCA) of the RNA-seq and microarray dataset for 26 liver samples. (1) Two-component PCA for Microarray dataset of ANIT, MDA, and CCL_4_ (left) (2) RNA-Seq dataset of ANIT, MDA, and CCl_4_ (right). Percentages represent variance captured by each principal components 1 and 2 in each analysis. Controls are shown in green color circle and hepatotoxicants are colored differently. **(B)** Principal component analysis of APAP and DCLF liver samples (1) two-component analysis of microarray data for APAP and DCLF (left). The beige color represents water treated control samples. The red colored samples are DCLF treated. The light and dark green circles represent corn oil control and APAP treated samples respectively. (2) RNA-Seq data analysis on APAP and DCLF (right). The drug treated samples are shown within the closed circle or oval shaped ring.

In toxicogenomic studies, PCA is generally used to analyze the complex multi-dimensional gene expression datasets. PCA results for the RNA-Seq and microarray datasets are summarized in Figures 1A,B. The first and second principal components (PC1 and PC2) accounted for 30 and 12% variability in the RNA-Seq dataset (i.e., matrix of 32,663 transcripts X 26 samples). A similar assessment of the microarray dataset (i.e., matrix of 21,419 X 26 samples) showed a smaller degree of separation (18 and 13%). A clear segregation of ANIT-, MDA-, CCl_4_-, and DCLF-treated samples from their corresponding controls was observed in both the RNA-Seq and microarray datasets (Figures 1A,B), reflecting the differential gene expression patterns observed with these hepatotoxicants. APAP-treated samples did not separate from their respective control samples with either microarray or RNA-Seq, reflecting the low level of gene expression changes, which was highly consistent with the lack of observed histopathological and serum chemistry findings (Supplementary Figures S1A–E and Supplementary Table S2). The computed additional principal components, such as PC3 to PC10 for APAP on both RNA-Seq and microarray data also provided similar inseparable results (for example, PC3 7.69%, PC4 4.49%, PC5 3.56%, PC6 3.2%, PC7 3.2%, PC8 2.8%, PC9 2.7%, and PC10 2.7%).

### Absolute Gene Expression Concordance Between the RNA-Seq and Microarray Platforms

**FIGURE 2:**
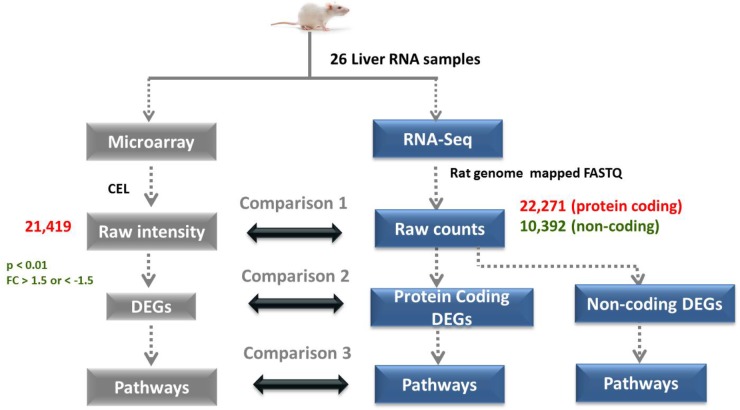
Overall computational process of RNA-Seq and microarray data analysis. 26 liver samples (15 drug treated and 11 controls) were assessed by microarray and RNA-Seq platform. Comparison at raw expression, differentially expression and pathway stages are indicated. A statistical criteria of *p* < 0.01 and FC < –1.5 or FC > 1.5 were used to obtain DEGs from raw expression data.

**FIGURE 3:**
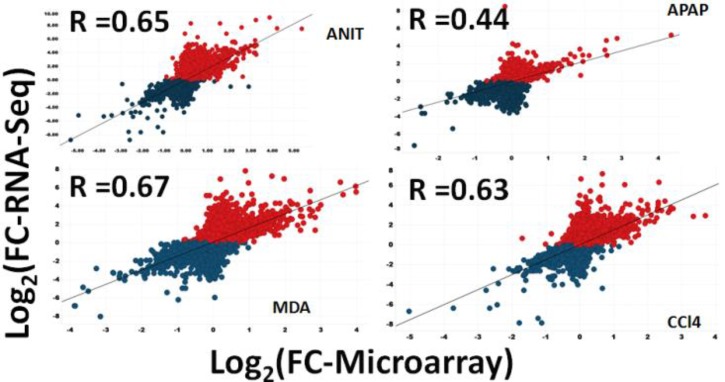
Scatter plot showing the relative expression levels of genes in terms of log_2_FCs for 18,776 consensus genes, determined by RNA-Seq and microarray. Log_2_FC is computed by taking average of three samples. Blue indicates RNA-Seq’s down-regulated and red is up-regulated protein coding genes. The graphs show that the overall FC dynamic ranges (log2 transformed) for 18,776 genes.

To identify a general linear relationship between the RNA-Seq counts to the corresponding microarray fluorescence intensities for all the expressed genes, a Spearman’s correlation coefficient was computed to check the data consistency between the two platforms (Figure 2, Comparison 1). A quantitative comparison of the relative raw expression profile of the 18,776 genes present in both platforms is shown in Figure 3 for livers treated with ANIT, APAP, MDA, DCLF, and CCl_4_. Encouragingly, the measured gene abundance derived from these two different gene expression methods showed a correlation of 0.65, 0.67, and 0.65 for ANIT, MDA, and CCl_4,_ respectively, indicating a good concordance between the platforms. However, the computed Spearman’s correlation for APAP and DCLF were 0.44 and 0.30, respectively, indicating a weak correlation between the platforms. An additional computational analysis on the APAP and DCLF gene expression datasets revealed the presence of significant variability for genes expressed at low absolute levels in the microarray platform, likely explaining, at least in part, the poorer correlation for these samples with minimal toxic changes (Supplementary Figures S2A,B).

### DEGs Concordance Between the RNA-Seq and Microarray Platforms

**FIGURE 4:**
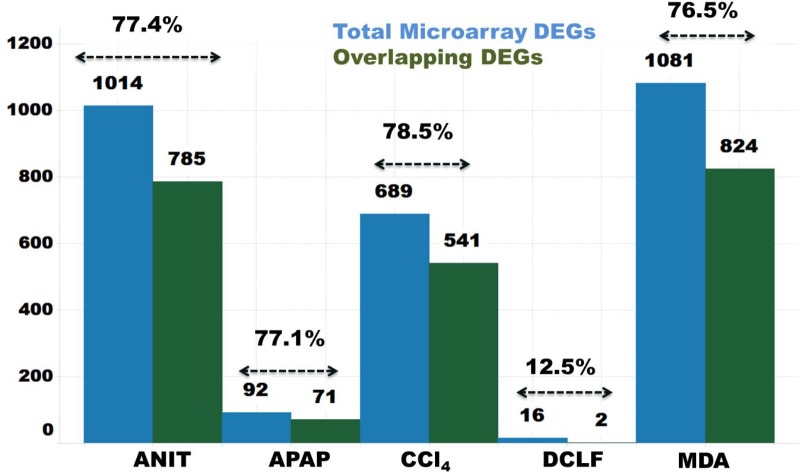
Concordance of protein-coding DEGs from RNA-Seq and Microarray. Blue and green bars indicate total number of microarray platform identified DEGs and number of inter-platform overlapping DEGs, respectively.

**FIGURE 5:**
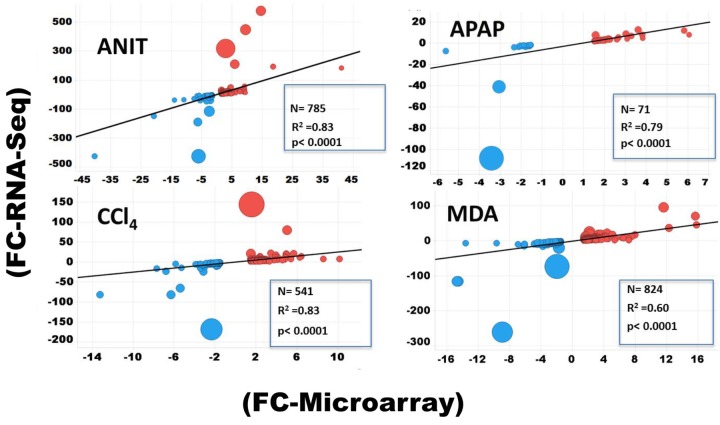
Spearman’s correlation plot for DEGs determined by RNA-Seq and microarray. Size of the filled circle is proportional to fold change difference (i.e., the larger the circle the bigger the FC difference). The blue and red spheres indicate down and upregulated DEGs, respectively.

The protein-coding DEGs identified by RNA-Seq and microarrays were compared (Figure 2, Comparison 2). Overall, the RNA-Seq platform captured a larger number of protein-coding DEGs compared to the microarray platform for all the tested hepatotoxicants (Table 1A: columns 4 and 7). The RNA-Seq platform identified a modulation of expression for 3139 (or 16.7% of the expressed protein-coding genes), 512 (2.7%), 3672 (19.5%), 2127 (9.5%), and 196 (1.0%) genes for ANIT, APAP, MDA, CCl_4_, MDA, and DCLF, respectively. In contrast, with the microarray platform, only 1014 (5.4% of the expressed protein-coding genes), 92 (0.4%), 1081 (5.7%), 689 (3.6%), and 16 (0.1%) of the total protein-coding sequences were differentially expressed for ANIT, APAP, MDA, CCl_4_, and DCLF, respectively. Hence for all tested hepatotoxicants, less than half of the DEGs were identified with microarrays compared to RNA-Seq. Of these, 785, 71, 824, 541, and 2 DEGs were also found to be differentially expressed in the RNA-Seq platform for ANIT, APAP, MDA, CCl_4_, and DCLF, respectively, resulting in an overlap of ∼78% between the two platforms except for DCLF (Figure 4 and Table 1B: column 4). The computed Spearman’s correlation for these overlapped DEGs of ANIT, APAP, MDA, and CCl_4_ were 0.83, 0.79, 0.70, and 0.83, respectively, revealing a significant concordance between the platforms. For more than 95% of these up- and down-regulated DEGs, both platforms showed a similar directionality in expression changes, further demonstrating the excellent concordance between the two platforms (Figure 5).

**Table 1a:** Summary of number of DEGs from RNA-Seq and Microarray.

Treatment	Microarray (up-regulated)	Microarray (down-regulated)	Microarray (total)	RNA-Seq (up-regulated)	RNA-Seq (down-regulated)	RNA-Seq (total)
ANIT	507	507	1014	1634	1505	3139
APAP	54	38	92	275	237	512
CCl_4_	361	328	689	1091	1036	2127
DCLF	3	13	16	93	103	196
MDA	569	512	1081	1899	1773	3672

**Table 1B:** Concordance of DEGs between RNA-Seq and microarray platforms.

Treatment	Microarray (total)	Overlapping DEGs with RNA-Seq	% of DEGs overlap
ANIT	1014	785	77
APAP	92	71	77
CCl_4_	689	541	78
DCLF	16	2	12
MDA	1081	824	76

**FIGURE 6:**
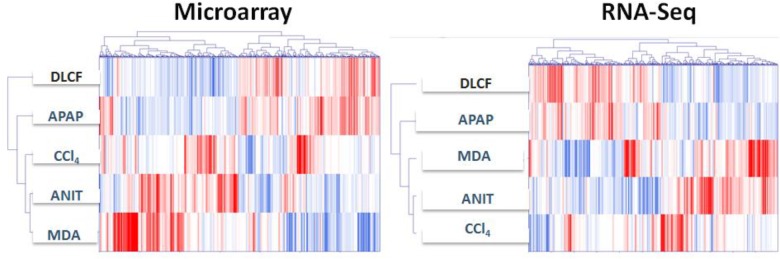
Hierarchically clustered genes (columns) and samples (rows) with dendrograms and clusters (blue colored bars). Red in the heatmap denotes upregulation while blue denotes downregulation.

The DEGs profiles in the ANIT, APAP, MDA, CCl_4_, and DCLF samples as detected by both platforms were also visually compared using a heatmap combined with two-way hierarchical clustering (Figure 6). The computed dendrogram shows three distinct groups for both microarray and RNA-Seq DEGs. In group 1, DEGs from the ANIT, MDA, and CCl_4_ samples clustered together, while the APAP and DCLF clustered separately in groups 2 and 3, consistent with the lack of obvious hepatotoxicity with these two toxicants. However, the DEGs from the ANIT and CCl_4_ samples were close to each other in group 1 with the RNA-Seq platform compared to the microarray platform, where the ANIT and MDA samples grouped together (Figure 6). This dendrogram also showed a comparable distribution of up- and down-regulated DEGs for ANIT, APAP, MDA, and CCl_4_ with both platforms, in spite of the fact that the set of DEGs that were up- and down-regulated with the two platforms was different.

Table 1C compares the dynamic range of expression for the DEGs detected with the two platforms and supports the better performance of the RNA-Seq platform for the identification of genes across a broad expression range. Particularly, the computed FC values for 239, 10, 134, and 86 DEGs of ANIT, APAP, MDA, and CCl_4_, respectively were at least two times higher with RNA-Seq compared to microarrays, confirming the higher sensitivity of the RNA-Seq platform. Furthermore, with microarrays, the FC saturated at the high end and increased background noise was noted at the low end. Overall, these results showed that RNA-Seq is better at capturing the gene expression changes for genes expressed at overall low levels and more precise in quantifying expression changes for the highly dysregulated genes.

**Table 1C:** Comparison of dynamic range of RNA-Seq and microarray.

Treatment	Microarray Max FC	Microarray Min FC	RNA-Seq Max FC	RNA-Seq Min FC
ANIT	41	−40.2	577.5	−884.9
APAP	20.3	−6.3	363.8	−156.4
CCl_4_	12.9	−14.1	143.8	−262
DCLF	2.1	−3.4	50.9	−28.3
MDA	15.7	−15.1	226.5	−260.5

### Unique Protein Coding DEGs Detected by the RNA-Seq and Microarray Platforms

Differentially expressed genes that are common to both platforms were subtracted from the total number of protein coding DEGs identified in each platform (Table 1D: columns 3 and 4). RNA-Seq identified at least 10 times more unique DEGs compared to microarrays for all the tested hepatotoxicants (Table 1D, column 5).

**Table 1D:** Summary of DEGs unique to RNA-Seq and microarray platforms.

Treatment	Overlapping DEGs between RNA-Seq and microarray	Microarray unique (total microarray – overlapping DEGs)	RNA-Seq unique (total protein coding RNA-Seq – overlapping DEGs)	RNA-Seq/microarray
ANIT	785	229	2354	10.2
APAP	71	21	441	21.0
CCl_4_	541	148	1586	10.7
DCLF	2	14	194	13.8
MDA	824	257	2842	11.0

RNA-Seq-specific DEGs are those that were shown to be differentially modulated only with the RNA-Seq platform. To obtain a complete perspective on these RNA-Seq specific protein-coding DEGs, a statistical criterion of *p* < 0.01 with higher FC such as ±1.5, 3, and 10 were used to generate a comprehensive list of DEGs (Table 2). ANIT, APAP, CCl_4_, MDA, and DCLF treatment differentially regulated 1367, 218, 835, 1621, and 193 protein-coding genes, translating to 7.2, 1.1, 4.4, 8.6, and 1% of the total expressed protein-coding genes (with FC ±1.5 criteria), respectively. Our analysis also identified a set of DEGs that were uniquely detected with microarrays. ANIT, APAP, MDA, and CCl_4_ treatment differentially regulated the expression of 229, 21, 257, and 148 genes, translating to 1.2, 0.1, 1.3, and 0.7% of the total expressed protein-coding genes, respectively (Supplementary Excel File S1). The computed FC for these genes generally ranged only from −1.5 to 2.

**Table 2:** Summary of RNA-Seq platform specific protein-coding DEGs.

Treatment	Total No. of DEG with FC > 1.5 or < −1.5	Dynamic range	Total No. DEG with FC > 3.0 or < −3.0	Total No. of DEG with FC > 10 or < −10
ANIT	1367	(**−**25.8,112.6)	459	77
APAP	218	(**−**12,1,293.7)	65	5
CCl_4_	835	(**−**16.1,100.7)	180	21
DCLF	193	(**−**28.3,50.9)	110	22
MDA	1621	(**−**62.5,122.1)	507	68

### Impacted Canonical Pathways by DEGs Identified With the RNA-Seq and Microarray Platforms

A major goal of this study was to understand whether the biology of the DEGs detected by each platform would lead to a similar understanding of the mechanism of toxicity (Figure 2, Comparison 3). Hence, the DEGs of APAP, ANIT, MDA, DCLF and CCl_4_, identified with either platform were analyzed with IPA to identify statistically significant canonical pathways. Supplementary Tables S3A–D summarizes the top impacted pathways identified by RNA-Seq and microarrays for ANIT, MDA, CCl_4_, and APAP treatments. Many of the top scoring canonical pathways detected by microarrays and RNA-Seq were similar for the most part and were relevant to liver toxicity. However, RNA-Seq DEGs uniquely captured a few additional liver relevant canonical pathways (Supplementary Tables S3A–D, shown in italics). Finally, it is reassuring that the DEGs of each tested hepatotoxicants detected with the two different gene expression platforms captured distinct liver-associated pathways for each drug, further confirming their distinct mechanism of toxicity.

The IPA Upstream Regulator analytic was also used to identify potential transcriptional regulators. Results showed, for example that both microarray- and RNA-Seq-derived DEGs with ANIT predicted a total of 113 (35 inhibition and 78 activation) and 73 (15 inhibition and 68 activation) regulators in RNA-Seq and microarray, respectively. Key notable high scoring regulators included (i) PPARα inhibition (2) OGA inhibition (3) let-7 (miRNA) inhibition (4) miR-21 inhibition and (5) AGT activation for the DEGs identified for ANIT in both platforms (Supplementary Table S4A). The top scoring regulators for MDA and CCl_4_ are listed in the Supplementary Tables S4B,C. For APAP, only NFE2L2 was predicted to be an upstream regulator. All the predicted upstream regulators (with z-score > 3 or < −3) for all the studied toxicants from microarray and RNA-Seq are listed in the Supplementary Excel Files S5A,B. Overall, these results indicate that the DEGs of RNA-Seq and microarray impacted similar upstream regulators. Additionally, the DEGs uniquely identified by RNA-Seq resulted in a few non-overlapping upstream regulators for the toxicants studied (Supplementary Excel File S6).

In a separate analysis, RNA-Seq and microarray specific DEGs (Table 1D- columns 3 and 4) were queried in IPA to identify statistically impacted canonical pathways. A large number of additional canonical pathways were identified by RNA-Seq alone (Supplementary Tables S5A,B). No additional statistically significant canonical pathways were identified by the microarray specific DEGs alone.

### Non-coding DEGs Detected by RNA-Seq

**FIGURE 7:**
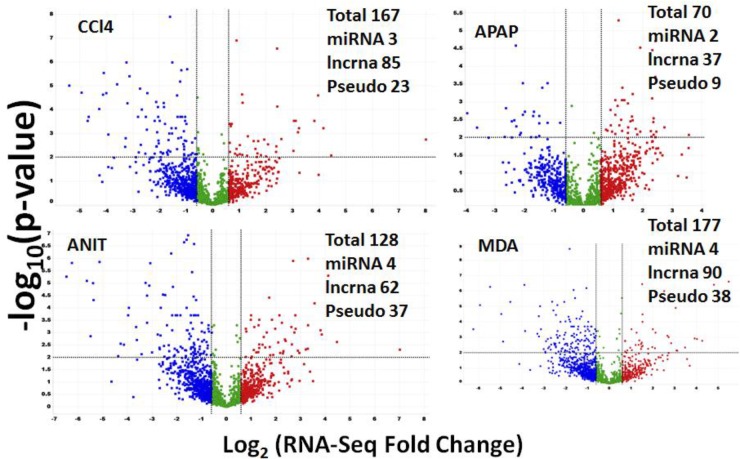
Volcano plot summarizing RNA-Seq specific non-coding DEGs. The red dots on the right top quadrant are significantly up-regulated non-coding DEGs and the dots within the top left quadrant shows highly down-regulated non-coding DEGs. Green color dots denote un-changed non-coding transcripts.

RNA-Seq can detect both protein-coding and non-coding DEGs in a single experiment. A total of 10,392 non-coding RNA transcripts were expressed in the rat liver samples. Of these, 3038 (29%), 1654 (15%), 1588 (15%), 1475 (14%), 803 (7%), and 612 (5.8%) transcripts were categorized as lncRNA, small nucleolar RNA (snoRNA), microRNA (miRNA), small nuclear RNA(snRNA), and pseudogene/processed pseudo genes, respectively. In total, 622 (i.e., 5.9% of the total expressed non-coding transcripts) were identified as differentially regulated by ANIT, APAP, CCl_4_, MDA, and DCLF combined (Figure 7). Table 3 summarizes the total number of non-coding DEGs along with the computed FC values for each category for each drug. APAP, ANIT, CCl_4_, MDA, and DCLF impacted the expression of 70, 167, 128, 177, and 80 non-coding genes, translating to 0.6, 1.6, 1.2, 1.7, and 0.7% of the total expressed non-coding transcriptome, respectively.

**Table 3:** Summary of RNA-Seq specific non-coding DEGs.

RNA-type	ANIT	APAP	CCl_4_	DCLF	MDA
lncRNA	85	37	62	26	90
Pseudogene	37	9	23	17	38
snRNA	4	3	4	9	1
miRNA	4	2	3	8	4
processed_pseudogene	9	5	13	8	15
snoRNA	9	4	5	6	2
rRNA	0	0	0	2	0
TEC	0	0	0	1	1
misc_RNA	4	3	3	1	3
processed_transcript	4	2	3	1	11
unprocessed_pseudogene	9	3	11	1	9
transcribed_processed_pseudogene	2	0	1	0	0
transcribed_unprocessed_pseudogene	0	0	0	0	2
Antisense	0	1	0	0	1
Ribozyme	0	1	0	0	0

Since the biology of lncRNAs is not clearly understood, we analyzed the *cis*-protein-coding DEGs of differentially expressed lncRNAs and inferred the potential biological insights for the significantly modulated lncRNAs. The closest protein-coding DEG to a differentially expressed lncRNA within the same chromosome is generally referred as the protein-coding “*cis*-gene” of a lncRNA. The highly impacted lncRNAs along with the nearest *cis*-DEGs for ANIT, APAP, MDA, CCl_4_, and DCLF are summarized in Supplementary Excel File S2. A total of 37, 62, 5, and 19 *cis*-DEGs were identified for ANIT, MDA, CCl_4_, and APAP, respectively (Supplementary Figures S3A–D and Supplementary Excel File S2). The FC values for most of the highly modulated lncRNAs positively correlated with the expression of *cis-*DEGs, indicating possible relationship between the lncRNA’s and its cognate protein-coding mRNAs.

A simple query within IPA of the ANIT impacted 37 *cis*-DEGs showed that (1) histidine degradation III, IV, (2) Biotin-carboxyl carrier protein assembly, (3) folate degradation and (4) protein citrulination are possible impacted canonical pathways, revealing possible mechanistic information related to ANIT toxicity with these pathways. Moreover, 20 of these *cis*-DEGs are associated with liver hyperplasia/hyperproliferation. Supplementary Excel File S3 summarizes the differentially regulated 21 miRNAs along with computed FC values. Of these, six miRNA’s have been annotated with liver biological function. The functionally annotated miRNAs include (1) mir3567 (MDA) and mir-6326 (MDA); (2) mir-let78 (DCLF); (3) mir3064 (APAP); (4) mir378b (ANIT); and (5) mir3791 (miR-122 family) (ANIT). The tested hepatotoxicants also differently modulated the expression of a total of 174 pseudogenes and processed pseudogenes, translating to 1.67% of the total differentially expressed non-coding transcripts (Supplementary Excel File S4).

## Discussion

The RNA-Seq and microarray platforms are fundamentally different from each other in terms of gene expression measurements. The former measures all RNA transcript counts, a direct measurement of gene expression, while the latter measures a fluorescence intensity that is due to hybridization with anti-sense probe sequences, an indirect measurement of gene expression. The advantage of RNA-Seq over microarrays is that it provides an unbiased insight into all transcripts (Zhao et al., 2014). Thus, RNA-Seq is generally reliable for accurately measuring gene expression level changes. Nevertheless, the key question is whether this improved reliability, accuracy, and sensitivity is sufficient to justify a switch from microarrays to RNA-Seq in the context of toxicogenomic studies.

Several studies have compared these two transcriptional profiling platforms for various purposes (Bottomly et al., 2011; van Delft et al., 2012; Merrick et al., 2013; Wang et al., 2014; Zhao et al., 2014). These studies overall indicate that: (1) a significantly larger number of DEGs and affected pathways can be detected with RNA-Seq compared to microarrays; (2) RNA-Seq has a wider dynamic range than microarrays; and (3) a reasonable concordance of DEGs (50–60%) exists between the two platforms. Our data are consistent with these studies in terms of overall acceptable concordance between the two platforms and regarding the higher sensitivity and better dynamic range observed with RNA-Seq. However, our study showed a higher level of concordance (>75%) between the two platforms compared to other studies. In addition, our study evaluated the potential utility of RNA-Seq for mechanistic toxicology investigations with more depth by using carefully selected hepatotoxicants with distinct mechanisms of toxicity and in a context relevant to the conduct of exploratory toxicology studies in the pharmaceutical industry.

There are at least three common approaches that map expression data from different platforms [RefSeq, Ensembl, University of California, Santa Cruz (UCSC)]. Additionally, some researchers align probe sequences to a recent release of the Genome or Transcriptome in an attempt to obtain the most up-to-date results. Many commercial vendors release updated annotation files (with varying degrees of regularity) in an attempt to keep these annotations current. Zhao and Zhang (2015) have comprehensively compared these different annotations within the context of the human genome. Abascal et al. (2018) have also compared the gene annotation from Ensembl, RefSeq, and UniprotKB and found an significant overlap of genes, suggesting annotations from different databases are somewhat in general agreement. Array Studio uses annotations from both Ensembl.R83 and RefSeq for gene identification from the microarray probes. For RNA-Seq transcripts, this tool uses Ensembl.R83 for gene identification. The identified genes from both platforms were used for comparison. It may be possible that a few genes have been missed by this annotation mismatch. However, within the context of toxicology studies, these annotation differences may play a limited role, as evidenced by the identification of similar biological pathways and upstream regulators identified with RNA-Seq and microarray DEGs.

An interrogation of the dynamic range values for the DEGs detected with the two platforms indicated that RNA-Seq has a dramatically larger dynamic range extending from 577- to −884-fold. In contrast, the dynamic range observed with microarrays ranged only from 41- to −40-fold. This appealing dynamic range feature of RNA-Seq effectively eliminated the saturation biases, which is inherent to microarray platforms. Consequently, RNA-Seq data have a tighter distribution of FC around 1.5, which drastically lowers the signal-to-noise ratios for genes expressed at low levels. Because of this sensitive nature, the RNA-Seq platform detected at least three times more protein-coding DEGs for all the hepatotoxicants compared to microarrays, in excellent agreement with reported platform comparison studies (Bohman et al., 2016). However, it should be noted that this observation is partly biased, since microarrays do not cover all possible cellular transcripts and since the gene coverage also differs across chip patterns.

The DEGs detected with RNA-Seq resulted in more significantly altered pathways compared to microarrays, which suggests that RNA-Seq provides more information about toxicant-induced transcriptomic perturbations. Nevertheless, there was also a significant overlap in the top modulated canonical pathways identified by the protein-coding DEGs of both platforms, and for the most part, the toxicological interpretation of these transcriptomic changes was quite similar, in agreement with observations by others (Su et al., 2011). However, the DEGs of RNA-Seq uniquely identified a few additional pathways compared to microarrays, demonstrating some additional benefits of RNA-Seq. For example, RNA-Seq-derived DEGs induced by ANIT treatment uniquely identified the PPARα/PXR pathway, in agreement with a previously reported study that revealed this connection (Cui et al., 2009).

Although pathway analyses using analytical tools like IPA help summarize and interpret the complex biology behind drug-induced transcriptomic perturbations, these analyses are also intrinsically biased by the published knowledgebase without consideration for potential institutional knowledge and omit alternate pathway routes for regulated DEGs. Pathway annotation is mostly a manual process and all genes and functional relationships are generally not yet fully covered. Moreover, the pathways are not universally defined and different tools identify different pathway results for the same datasets (Khatri et al., 2012). For example, in the current study, a significant number of highly regulated protein-coding DEGs identified with RNA-Seq and microarrays were not associated with any of the IPA annotated canonical pathways. Thus, the development of an analysis environment that exploits both canonical pathways and new extended network interactions may improve our understanding of the significance of highly regulated DEGs within the context of liver pathological processes (Cerami et al., 2010; Khatri et al., 2012). There has been tremendous progress in the pathway curation and integration process during the past few years and that progress has resulted in novel pathway tools (Fabregat et al., 2017; Huang et al., 2017; Uppal et al., 2017; Wang et al., 2017; Forsberg et al., 2018; Pittman et al., 2018; Ukmar et al., 2018). However, these tools are still highly fragmented and not integrated into a single framework for optimal DEGs analysis. Integration of multiple pathway analysis tools may be needed to better extract the comprehensive biological information present in the DEGs of RNA-Seq and microarrays.

An important advantage of RNA-Seq over microarrays is its ability to measure almost all types of RNAs in a single experiment. Recently, non-coding RNAs have generated significant interest in toxicological and biomarker research (Dempsey and Cui, 2016; Gong et al., 2016). These non-coding RNAs are not typically detected with standard microarray chips based on design, but can be captured and quantified by RNA-Seq. The RNA-Seq platform in our study uniquely identified a total of 622 differentially regulated non-coding transcripts for all toxicants combined (about 5.9% of the total expressed non-coding transcripts). These non-coding transcripts include miRNAs, miscRNAs and lncRNAs, pseudogenes, snRNAs, snoRNAs, and unprocessed transcripts. Despite this library preparation kit being optimized for mRNAs, we still detected these non-coding RNAs. This suggests that even more non-coding DEGs may be detected with an alternative library prep kit. The tested hepatotoxicants mainly impacted the expression of lncRNAs, pseudogenes and miRNAs and these have a potential for use as toxicity biomarkers and may offer additional mechanistic insight in some cases (Esteller, 2011; Ling et al., 2013).

Long non-coding RNA represent a new class of biologically important molecules with nucleotide length of > 200 bases (Kapranov et al., 2007). These non-coding RNAs are generally less stable and are expressed at lower levels compared to the protein coding mRNAs. The relative abundance of mRNAs is about 10 times greater than that of lncRNA (Djebali et al., 2012). Moreover, lncRNA expression is highly restricted to certain tissue types such as testis, heart, and liver (Derrien et al., 2012; Necsulea et al., 2014) and lncRNAs are localized within the chromatin compartment of the nucleus. Quantitatively, 80% of the expressed lncRNAs have been characterized as tissue specific, in contrast to <20% for mRNAs (Yan et al., 2013). The process of quantification of these low abundant tissue-specific lncRNA transcripts remains a challenging and on-going task.

Recent studies suggest that lncRNAs bind to chromatin, chromatin modifying proteins, certain transcription factors, and miRNAs. This binding event significantly regulates a wide range of mechanisms like epigenetic signaling, disrupting polymerase activities and altering miRNA stability (Baumgart et al., 2016). Additionally, it is now also well-accepted that lncRNAs are connected with various biological processes (Kung et al., 2013) and diseases (Schmitt and Chang, 2016; Hon et al., 2017). Thus, lncRNAs have been recognized as potential markers for liver injury (Takahashi et al., 2014) and could serve as potential toxicity biomarkers.

Long non-coding RNAs are generally identified using three established criteria, namely: (1) lack of an open reading frame; (2) sequence size of <200 bases; and (3) poor homology with sequences of known proteins (Jalali et al., 2015). Our study uniquely identified a total of 300 differentially regulated lncRNAs across all toxicants combined. Almost 50% of these lnRNAs displayed a large change in expression level (FC of −50 to 85 with *p* < 0.01). Although the biological function of these highly modulated lncRNAs is unclear, there have been a number of reports (Zhu et al., 2014) indicating a positive correlation between the expression of lncRNAs to the nearest protein-coding genes within the same chromosome as confirmed by our analysis. For example, ANIT upregulated the expression of AABR7003056.1 with a FC value of 3.09. The *cis*-gene of this lncRNA ccnE1 was also upregulated with a FC value of 7.9. Increased expression of ccnE1 has been reported in human and mouse liver fibrosis (Nevzorova et al., 2012). Perhaps the high expression of ccnE1 may have contributed to the observed liver effects for ANIT.

miRNAs are small non-coding RNA molecules (containing about 22 bases) that function in RNA silencing and post-transcriptional regulation of gene expression. While the majority of miRNAs are located within the cell, some miRNAs, commonly known as circulating or extracellular miRNAs, have also been found in the extracellular environment, including various biological fluids (Wang et al., 2009). During the past decade, miRNAs have generated a high level of interest in toxicology (Clarke et al., 2014; Krauskopf et al., 2015). miRNAs are not typically detected with standard microarray chips but can be identified and quantified as part of a standard RNA-Seq analysis. Our study identified a total of 21 differentially regulated miRNAs across all toxicants combined. For example, ANIT down-regulated miR3591 and miR378b (miR122 family) in rat liver. ANIT treatment in mice for 48 h has been shown to reduce the expression of hepatocyte nuclear factor 1-alpha (Hnf1a) (Tanaka et al., 2009), consistent with our study where Hnf1a was down-regulated by both RNA-Seq and microarrays. Interestingly, miR3591 down-regulation correlates with *Hnf1a* gene down-regulation (Coulouarn et al., 2009) and this is also consistent with our study that showed miR3591 down-regulation.

Psesudogenes are generally produced through a wide range of mechanisms (Zhang et al., 2010). A spontaneous mutation in a protein-coding gene can generally prevent either transcription or translation of the gene, resulting in the formation of unitary pseudogene. Additionally, duplicated pseudogenes are also generated through a tandem doubling of certain sequences. These duplicated and unitary pseudogenes lose their protein-coding capability due to either the loss of promoters or mutations that create premature stop codons (Mighell et al., 2000) However, these pseudogene sequences are released from selection pressure and accumulate as non-gene-like features. These accumulated pseudogene sequences and their cognate protein coding genes form regulatory pairs that control each other’s activities (Kalyana-Sundaram et al., 2012). For example, knock-down of pseudogene ABCC6P1 has been shown to decrease the expression of its cognate protein-coding gene ABC66 (Pink et al., 2011), suggesting that pseudogenes can exert regulatory effects on their protein coding genes. The co-expression of pseudogenes and their cognate protein-coding genes have not been looked at thoroughly within the context of toxicity assessment in a single experiment, as pseudogenes probes are generally absent from typical microarray chips. Our RNA-Seq data identified a total of 174 pseudogenes with altered expression from all toxicants combined. The identification of a possible link between liver toxicity to these non-coding pseudogene/protein-coding gene regulatory pairs may give us an additional mechanistic insight into the roles of some of the highly impacted pseudogenes. Altogether, although it is premature to draw conclusions, it appears that measurement of non-protein-coding transcripts (lncRNAs, miRNAs, and pseudogenes) may provide some useful insights regarding mechanisms of liver toxicity. Future *in vitro* and *in vivo* studies are clearly necessary to further understand the utility for mechanistic molecular toxicology of these non-protein-coding diagnostic and prognostic transcripts.

Microarrays measure the expression of only pre-defined probes (genes) and typical arrays are designed to cover only a portion of protein-coding genes. Thus, it is currently impossible to detect regulation of non-coding genes (i.e., lncRNAs, miRNAs, and pseudogenes), other important novel RNAs and biologically relevant novel splice variants in addition to the complete protein-coding transcriptome in a single array experiment. Furthermore, hybridization can result in mismatch between probes and target molecules, leading to increased noise and higher likelihood of misidentified DEGs. Because of its added advantages, RNA-Seq is progressively replacing microarray technology for many transcriptomic applications (Lowe et al., 2017). However, microarrays still offer some advantages. Firstly, microarray data are more manageable in size: the size of RNA-Seq datasets is generally about >50 times larger depending on the sample size and sequencing depth. In the present study, we generated 39 and 0.5 GB of RNA-Seq and microarray data from the 26 samples, respectively, a difference of 78-fold in file size. Even for this simple prototype study, this massive amount of data introduced data management and analysis challenges. Secondly, the overall computation time, data storage and management time for a microarray experiment are much lower. Based on our experience, to completely process and summarize the DEGs from a set of microarray-generated gene expression data generally take hours, depending on the amount of transcriptional change in the experiment. Thirdly, a large number of toxicogenomic studies have been conducted during the past two decades in various R&D organizations, thereby generating large microarray-based transcriptomic datasets. These datasets complemented by public databases such as GEO, DrugMatrix and Array Express have created easily accessible and analyzable databases, which serve as a critical reference for new toxicogenomic data analytics and interpretation. In contrast, there are no such reference databases available for RNA-Seq data, which currently limits toxicogenomic data interpretation. There is a clear need to build these databases to better leverage microarray data to facilitate RNA-Seq data interpretation and to enable the seamless translation/comparison of RNA-Seq data and microarray data in the context of toxicogenomic studies. Fourthly, data processing and analyses are well-established with microarrays; in contrast, as RNA-Seq is still new and evolving, there is not yet a single standardized computational approach for performing an RNA-Seq data analysis. However, with the recent advancement in computing power, hardware and dedicated computational workflows, this limitation will become rapidly obsolete. Finally, cost has probably been an important consideration for not switching from microarrays to RNA-Seq for toxicogenomic studies. However, based on the current study, RNA-Seq data generation was about 1.5 times less expensive than with microarrays. Taken together, these findings suggest that RNA-Seq should provide a comprehensive picture of protein-coding and non-coding DEGs as well as a more complete list of impacted canonical pathways at a lesser cost than microarrays.

## Conclusion

The present study indicates that RNA-Seq is a good alternative to microarrays for toxicogenomic studies of rat liver. In addition of detecting the majority of trancriptomic perturbations observed with microarrays, RNA-Seq captured additional DEGs and canonical pathways relevant to liver toxicity. The wider dynamic range offered by RNA-Seq provides a higher level of sensitivity and accuracy, as well as the ability to detect expression changes in non-coding genes that may offer important new insights into xenobiotic-induced liver toxicity. Given the critical role of databases for the accurate interpretation of toxicogenomics studies and the fact that institutional and public databases are largely based on microarray data, generation of RNA-Seq-based databases and better translation of microarray databases for comparison to and interpretation of RNA-Seq data are needed. However, the improved sensitivity, accuracy and ability to evaluate non-coding genes of RNA-Seq may prove valuable for studies designed to investigate mechanisms of toxicity.

## Author Contributions

All authors are aware of the manuscript and have contributed significantly to its completion. In addition, all authors are employed at AbbVie and the appropriate disclosures are included on the title page along with keywords.

## Conflict of Interest Statement

All authors are employees of AbbVie. The design, study conduct, and financial support for this research was provided by AbbVie. AbbVie participated in the interpretation of data, review, and approval of the publication. The reviewer SA and handling Editor declared their shared affiliation.

## Supplementary Material

FIGURE S1 **(A)** ANIT and **(B)** MDA administration resulted in bile duct epithelial hyperplasia and hypertrophy with infiltration of neutrophils and mononuclear into periportal spaces. Neutrophils are particularly prominent in MDA toxicity, with many neutrophils in the bile duct lumina. The inset images demonstrate the cytologic features of bile duct epithelial hypertrophy. **(C)** CCl_4_ administration resulted in macrovesicular and microvesicular hepatocellular steatosis with a centrilobular distribution. The inset image demonstrates a vacuolated hepatocyte. **(D)** Diclofenac and **(E)** APAP, administration under the dosing regimen described did not result in histopathological evidence of hepatocellular or bile duct injury. The extent of vacuolization observed in some hepatocytes was not significantly different from untreated control. Insets for **(D,E)** demonstrate individual hepatocytes with cytoplasmic features not different from vehicle control. In each panel, the scale bar represents 100 μm for the large figure and 10 μm for the inset.

FIGURE S2 **(A,B)** Fold change variation between RNA-Seq and Microarray for low abundant genes of DCLF. **(A)** DEGs of DCLF: blue color bar denotes FC from microarray and red color shows FC difference between RNA-Seq and microarray. **(B)** Comparison of raw data of DCLF from RNA-Seq and Microarray. The red color line shows the genes that are highly regulated in RNA-Seq compared to microarray. The green line shows genes with comparable expression in both platforms.

FIGURE S3 Significantly modulated *cis-*genes of **(A)** ANIT **(B)** MDA **(C)** CCl_4_ and **(D)** APAP. X-axis denotes *cis*-gene name and Y-axis is log_2_ transformed fold change value.

TABLE S1 Summary of RNA-Seq alignment statistics.

TABLE S2 Summary of serum chemistries.

TABLE S3 **(A)** ANIT impacted canonical pathways. The second column is computed −log (*p*-value) for RNA-Seq and the third column is for microarray. The RNA-Seq and microarray specific pathways are shown in italics. **(B)** APAP impacted canonical pathways. The second column is computed −log (*p*-value) for RNA-Seq and the third column is for microarray. The RNA-Seq and microarray specific pathways are shown in italics. **(C)** MDA impacted canonical pathways. The second column is computed −log (*p*-value) for RNA-Seq and the third column is for microarray. The RNA-Seq and microarray specific pathways are shown in italics. **(D)** CCl_4_ impacted canonical pathways. The second column is computed −log (*p*-value) for RNA-Seq and the third column is for microarray. The RNA-Seq and microarray specific pathways are shown in italics.

TABLE S4 **(A–C)** Upstream regulator analysis using Ingenuity Pathway Analysis. Top upstream regulators predicted based on the genes that were significantly different for **(A)** ANIT, **(B)** MDA, and **(C)** CCl_4_ and MDA treatments in RNA-Seq and microarray platforms are shown in column 1. The computed activation score and *p*-value for microarray and RNA-Seq are given in columns 2–3 and 4–6, respectively.

TABLE S5 **(A)** Pathways impacted by RNA-Seq specific DEGs of ANIT and APAP. Columns 1 and 2 corresponds to ANIT and third and fourth for APAP. **(B)** Pathways impacted by RNA-Seq specific DEGs of CCl_4_ and MDA. Columns 1 and 2 corresponds to CCl_4_ and third and fourth for MDA.

FILE S1 Summary of microarray specific DEGs.

FILE S2 Summary of differentially regulated lncRNAs and *cis*-DEGs.

FILE S3 Summary of differentially regulated miRNAs.

FILE S4 Summary of differentially regulated pseudogenes.

FILE S5 Top scoring upstream regulators for DEGs of **(A)** RNA-Seq and **(B)** microarray.

FILE S6 Summary of upstream regulators for RNA-Seq specific DEGs.
