# Supplementary figures and images for: Comparison of RNA-Seq and Microarray Gene Expression Platforms for the Toxicogenomic Evaluation of Liver From Short-Term Rat Toxicity Studies
Source: Front Genet. 2019 Jan 22;9:636. doi: 10.3389/fgene.2018.00636 (PMC6349826; doi:10.3389/fgene.2018.00636)

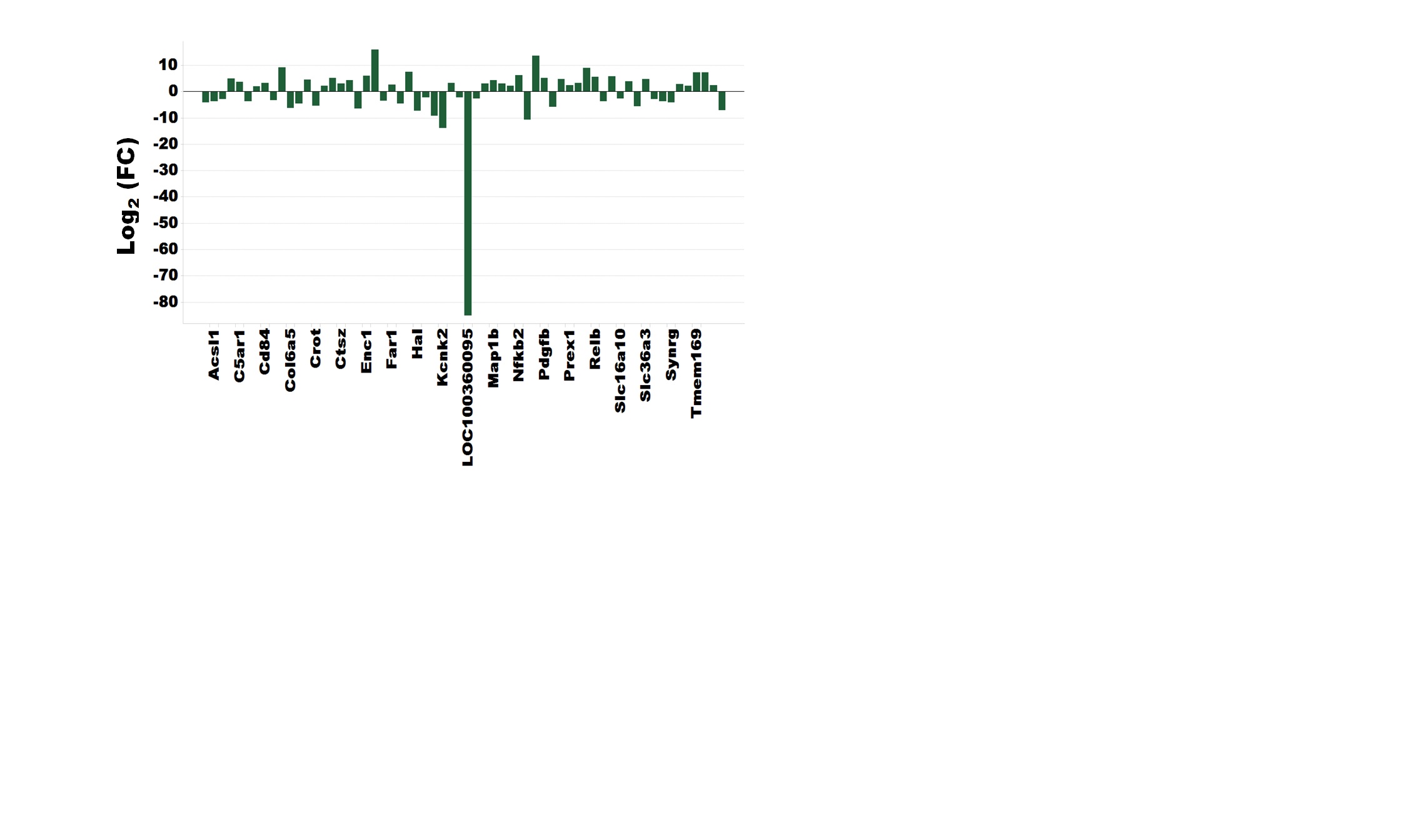

Supplement: FIGURE S1 — (A) ANIT and (B) MDA administration resulted in bile duct epithelial hyperplasia and hypertrophy with infiltration of neutrophils and mononuclear into periportal spaces. Neutrophils are particularly prominent in MDA toxicity, with many neutrophils in the bile duct lumina. The inset images demonstrate the cytologic features of bile duct epithelial hypertrophy. (C) CCl4 administration resulted in macrovesicular and microvesicular hepatocellular steatosis with a centrilobular distribution. The inset image demonstrates a vacuolated hepatocyte. (D) Diclofenac and (E) APAP, administration under the dosing regimen described did not result in histopathological evidence of hepatocellular or bile duct injury. The extent of vacuolization observed in some hepatocytes was not significantly different from untreated control. Insets for (D,E) demonstrate individual hepatocytes with cytoplasmic features not different from vehicle control. In each panel, the scale bar represents 100 μm for the large figure and 10 μm for the inset. [file Data_Sheet_2.zip › Supplemental_Figure S3B.jpg]

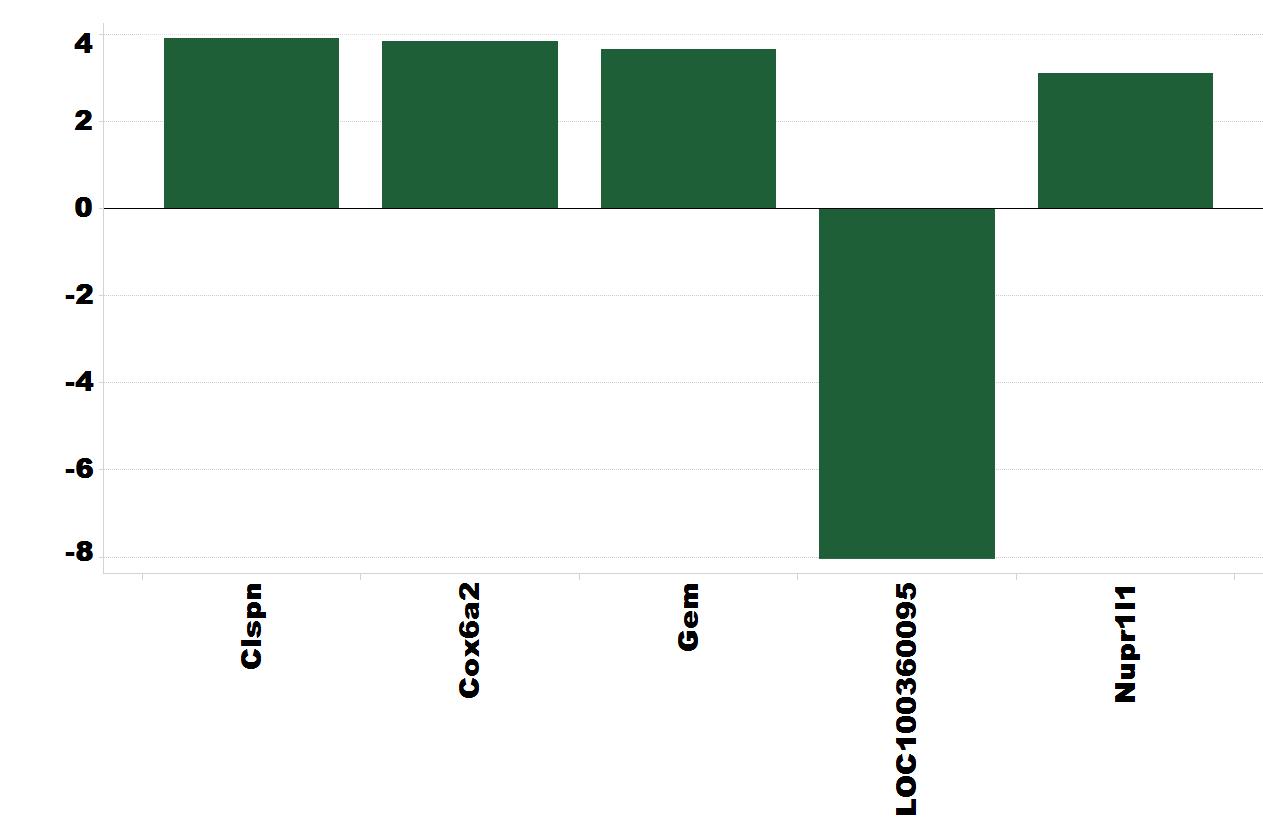

Supplement: FIGURE S1 — (A) ANIT and (B) MDA administration resulted in bile duct epithelial hyperplasia and hypertrophy with infiltration of neutrophils and mononuclear into periportal spaces. Neutrophils are particularly prominent in MDA toxicity, with many neutrophils in the bile duct lumina. The inset images demonstrate the cytologic features of bile duct epithelial hypertrophy. (C) CCl4 administration resulted in macrovesicular and microvesicular hepatocellular steatosis with a centrilobular distribution. The inset image demonstrates a vacuolated hepatocyte. (D) Diclofenac and (E) APAP, administration under the dosing regimen described did not result in histopathological evidence of hepatocellular or bile duct injury. The extent of vacuolization observed in some hepatocytes was not significantly different from untreated control. Insets for (D,E) demonstrate individual hepatocytes with cytoplasmic features not different from vehicle control. In each panel, the scale bar represents 100 μm for the large figure and 10 μm for the inset. [file Data_Sheet_2.zip › Supplemental_Figure_S3C.jpg]

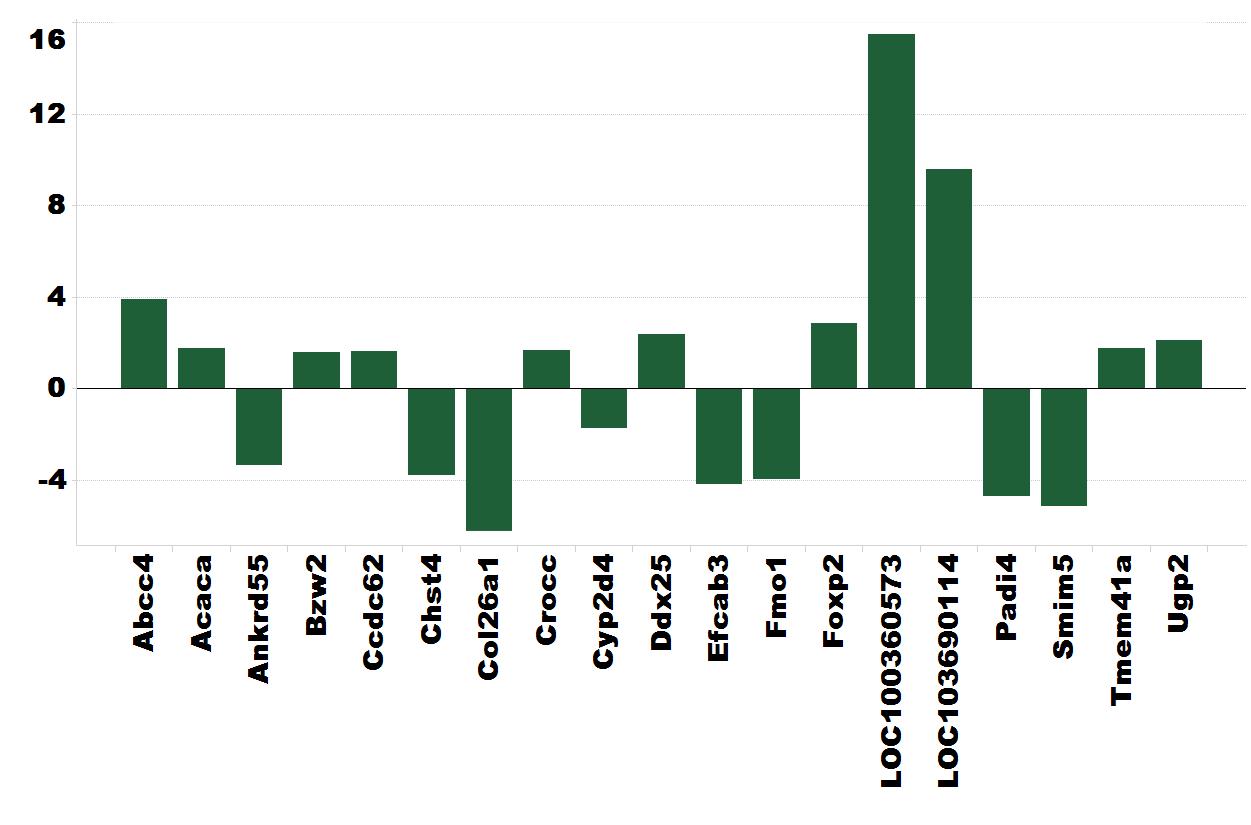

Supplement: FIGURE S1 — (A) ANIT and (B) MDA administration resulted in bile duct epithelial hyperplasia and hypertrophy with infiltration of neutrophils and mononuclear into periportal spaces. Neutrophils are particularly prominent in MDA toxicity, with many neutrophils in the bile duct lumina. The inset images demonstrate the cytologic features of bile duct epithelial hypertrophy. (C) CCl4 administration resulted in macrovesicular and microvesicular hepatocellular steatosis with a centrilobular distribution. The inset image demonstrates a vacuolated hepatocyte. (D) Diclofenac and (E) APAP, administration under the dosing regimen described did not result in histopathological evidence of hepatocellular or bile duct injury. The extent of vacuolization observed in some hepatocytes was not significantly different from untreated control. Insets for (D,E) demonstrate individual hepatocytes with cytoplasmic features not different from vehicle control. In each panel, the scale bar represents 100 μm for the large figure and 10 μm for the inset. [file Data_Sheet_2.zip › Supplemental_Figure_S3D.jpg]

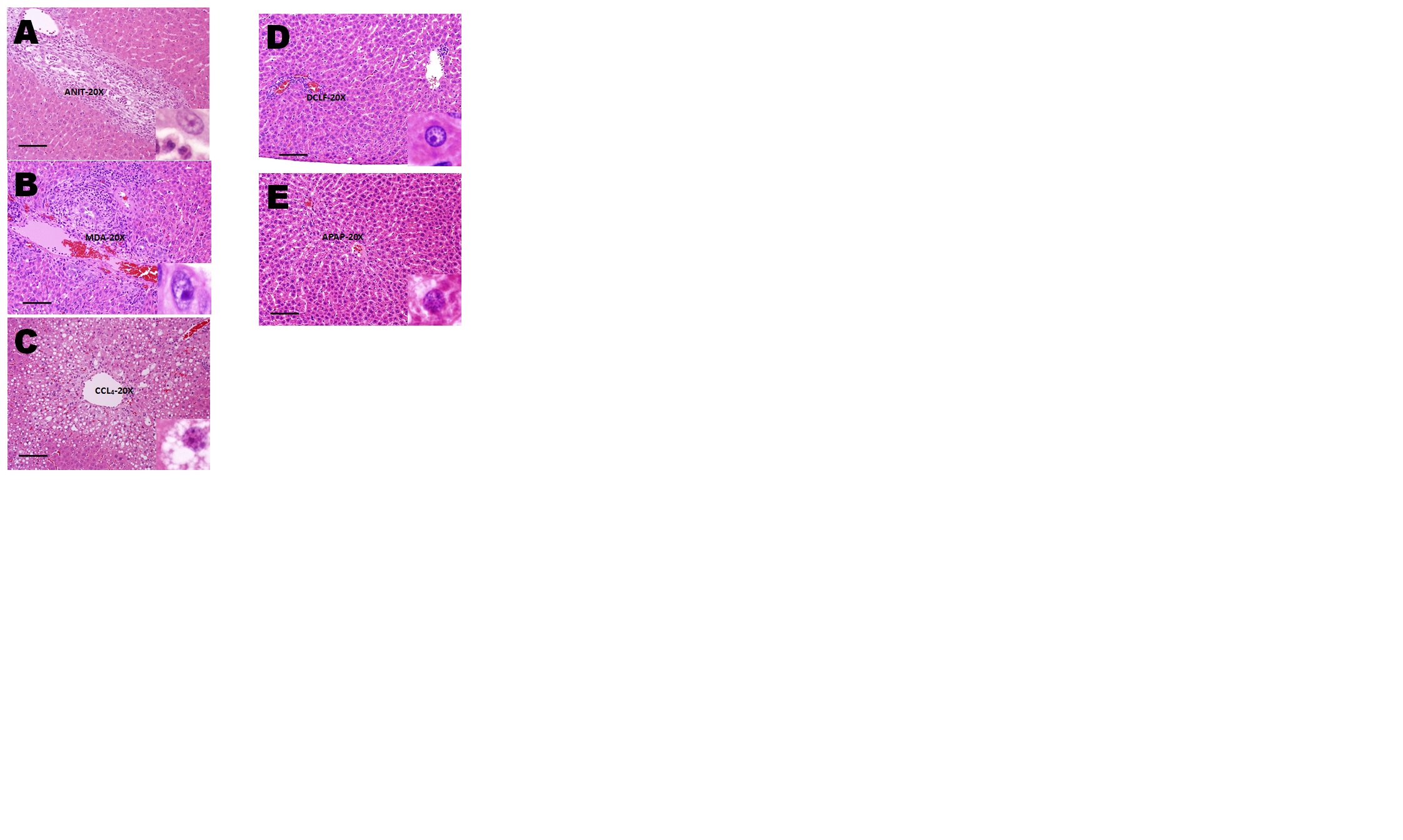

Supplement: FIGURE S1 — (A) ANIT and (B) MDA administration resulted in bile duct epithelial hyperplasia and hypertrophy with infiltration of neutrophils and mononuclear into periportal spaces. Neutrophils are particularly prominent in MDA toxicity, with many neutrophils in the bile duct lumina. The inset images demonstrate the cytologic features of bile duct epithelial hypertrophy. (C) CCl4 administration resulted in macrovesicular and microvesicular hepatocellular steatosis with a centrilobular distribution. The inset image demonstrates a vacuolated hepatocyte. (D) Diclofenac and (E) APAP, administration under the dosing regimen described did not result in histopathological evidence of hepatocellular or bile duct injury. The extent of vacuolization observed in some hepatocytes was not significantly different from untreated control. Insets for (D,E) demonstrate individual hepatocytes with cytoplasmic features not different from vehicle control. In each panel, the scale bar represents 100 μm for the large figure and 10 μm for the inset. [file Data_Sheet_2.zip › Supplemental_Figure S1A-E.jpg]

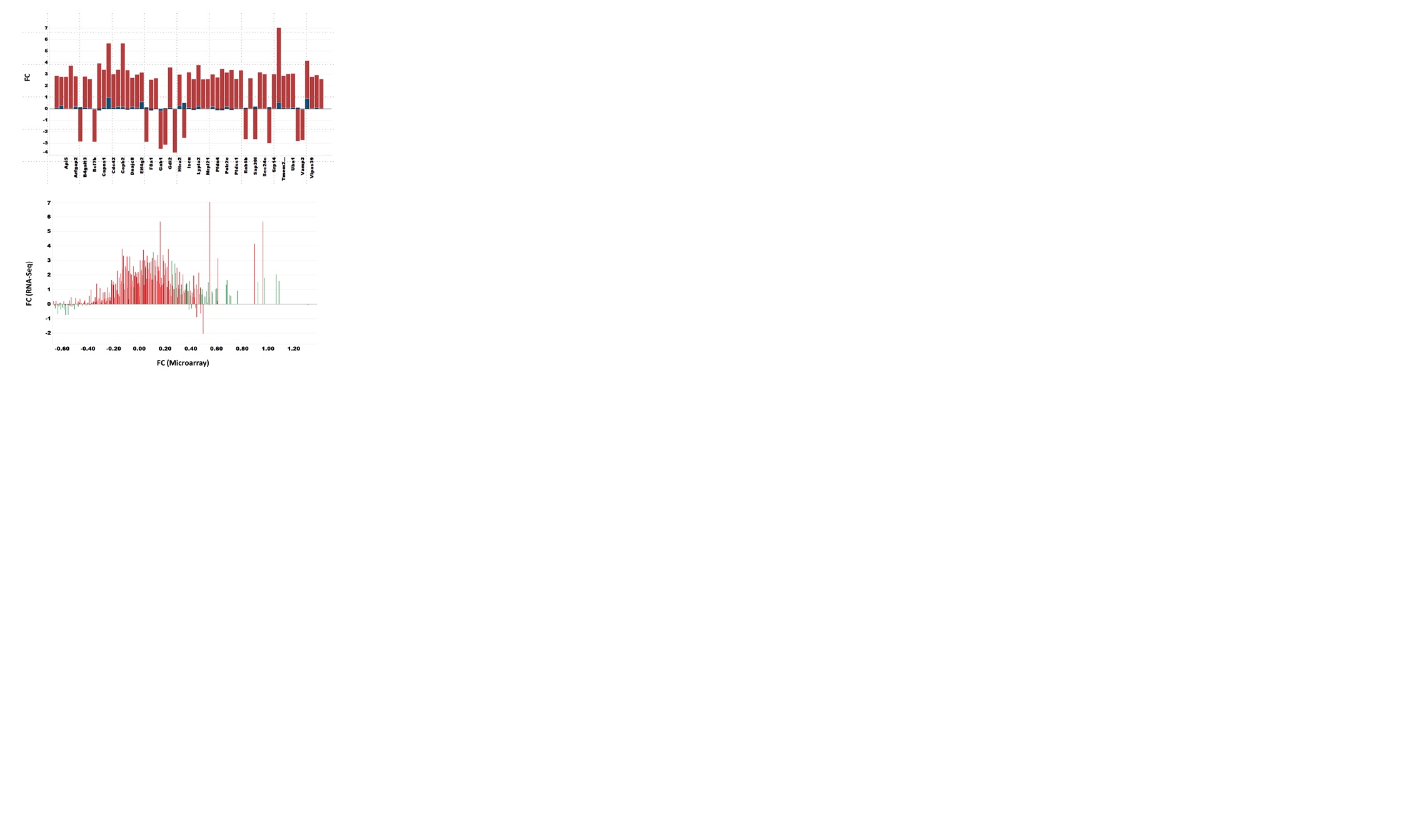

Supplement: FIGURE S1 — (A) ANIT and (B) MDA administration resulted in bile duct epithelial hyperplasia and hypertrophy with infiltration of neutrophils and mononuclear into periportal spaces. Neutrophils are particularly prominent in MDA toxicity, with many neutrophils in the bile duct lumina. The inset images demonstrate the cytologic features of bile duct epithelial hypertrophy. (C) CCl4 administration resulted in macrovesicular and microvesicular hepatocellular steatosis with a centrilobular distribution. The inset image demonstrates a vacuolated hepatocyte. (D) Diclofenac and (E) APAP, administration under the dosing regimen described did not result in histopathological evidence of hepatocellular or bile duct injury. The extent of vacuolization observed in some hepatocytes was not significantly different from untreated control. Insets for (D,E) demonstrate individual hepatocytes with cytoplasmic features not different from vehicle control. In each panel, the scale bar represents 100 μm for the large figure and 10 μm for the inset. [file Data_Sheet_2.zip › Supplemental_Figure S2A_B.jpg]

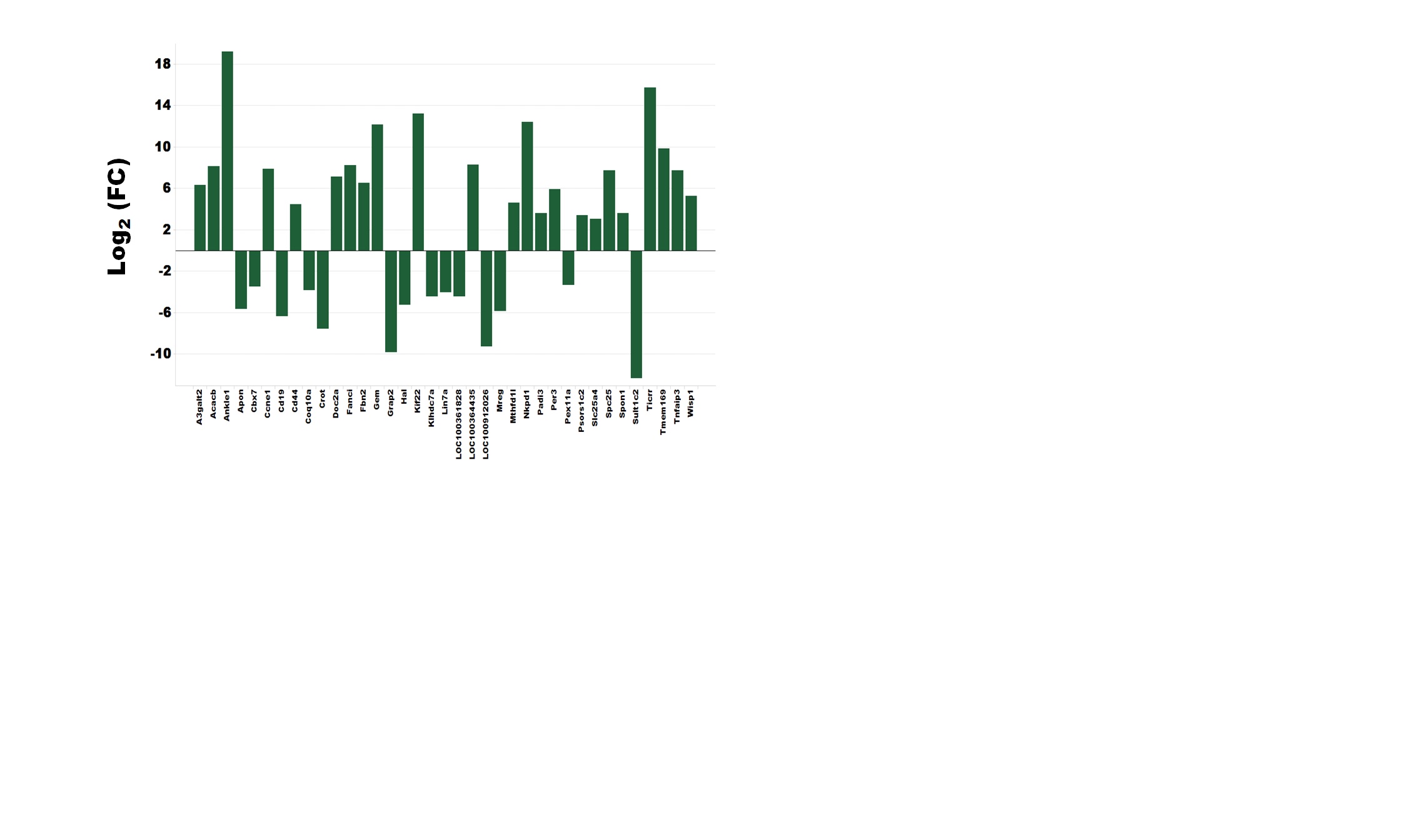

Supplement: FIGURE S1 — (A) ANIT and (B) MDA administration resulted in bile duct epithelial hyperplasia and hypertrophy with infiltration of neutrophils and mononuclear into periportal spaces. Neutrophils are particularly prominent in MDA toxicity, with many neutrophils in the bile duct lumina. The inset images demonstrate the cytologic features of bile duct epithelial hypertrophy. (C) CCl4 administration resulted in macrovesicular and microvesicular hepatocellular steatosis with a centrilobular distribution. The inset image demonstrates a vacuolated hepatocyte. (D) Diclofenac and (E) APAP, administration under the dosing regimen described did not result in histopathological evidence of hepatocellular or bile duct injury. The extent of vacuolization observed in some hepatocytes was not significantly different from untreated control. Insets for (D,E) demonstrate individual hepatocytes with cytoplasmic features not different from vehicle control. In each panel, the scale bar represents 100 μm for the large figure and 10 μm for the inset. [file Data_Sheet_2.zip › Supplemental_Figure S3A.jpg]
