# Supplementary material for: Comparison of RNA-Seq and Microarray Gene Expression Platforms for the Toxicogenomic Evaluation of Liver From Short-Term Rat Toxicity Studies
Source: Front Genet. 2019 Jan 22;9:636. doi: 10.3389/fgene.2018.00636 (PMC6349826; doi:10.3389/fgene.2018.00636)
Supplement: TABLE S1 — Summary of RNA-Seq alignment statistics. [file Data_Sheet_3.zip › Supplemental_Tables S1.docx]

**Table-S1: Summary of RNA-Seq alignment statistics**

| **Treatment** | **Total Reads (in million)** | **Uniquely Mapped**  **%** | **Non-Uniquely Mapped**  **%** | **Unmapped**  **%** |
| --- | --- | --- | --- | --- |
| **ANIT** | **22.49291** | **89.8937** | **6.7264** | **3.3799** |
| **ANIT** | **22.393758** | **91.0423** | **5.5987** | **3.3589** |
| **ANIT** | **20.96917** | **90.5668** | **5.9509** | **3.4823** |
| **APAP** | **31.105789** | **88.1236** | **7.4945** | **4.3819** |
| **APAP** | **30.523475** | **87.9967** | **7.1386** | **4.8647** |
| **APAP** | **27.420817** | **88.1967** | **7.5197** | **4.2836** |
| **CCl4** | **23.763074** | **88.6818** | **7.4304** | **3.8878** |
| **CCl4** | **22.756447** | **87.5154** | **6.8524** | **5.6323** |
| **CCl4** | **22.519095** | **89.7335** | **6.898** | **3.3686** |
| **DCLF** | **29.820898** | **87.8152** | **7.8486** | **4.3362** |
| **DCLF** | **28.551528** | **88.9046** | **6.9993** | **4.0961** |
| **DCLF** | **27.591556** | **87.9115** | **7.8109** | **4.2776** |
| **MDA** | **32.970728** | **88.0684** | **6.9638** | **4.9678** |
| **MDA** | **27.979772** | **88.8731** | **6.7931** | **4.3338** |
| **MDA** | **27.367709** | **88.7812** | **6.6597** | **4.5591** |
