# Supplementary material for: Comparison of RNA-Seq and Microarray Gene Expression Platforms for the Toxicogenomic Evaluation of Liver From Short-Term Rat Toxicity Studies
Source: Front Genet. 2019 Jan 22;9:636. doi: 10.3389/fgene.2018.00636 (PMC6349826; doi:10.3389/fgene.2018.00636)
Supplement: TABLE S1 — Summary of RNA-Seq alignment statistics. [file Data_Sheet_3.zip › Supplemental_TableS3A_D.docx]

**Supplemental Table S3A:**  ANIT impacted canonical pathways. The second column is computed –log (p-value) for RNA-Seq and the third column is for microarray. The RNA-Seq and microarray specific pathways are shown in italics

|  | | **-log_10_(p-value)** | **-log_10_(p-value)** |  |  |
| --- | --- | --- | --- | --- | --- |
| **RNA-Seq and microarray DEGs impacted common pathways** | | | |  |  |
| Superpathway of Cholesterol Biosynthesis | 15.5 | | 20.9 |  |  |
| LPS/IL-1 Mediated Inhibition of RXR Function | 10.4 | | 12.5 |  |  |
| Cholesterol Biosynthesis I | 10.1 | | 10.6 |  |  |
| Cholesterol Biosynthesis II (via 24,25-dihydrolanosterol) | 10.1 | | 10.6 |  |  |
| Cholesterol Biosynthesis III (via Desmosterol) | 10.1 | | 10.6 |  |  |
| Acetone Degradation I (to Methylglyoxal) | 6.83 | | 5.82 |  |  |
| Bupropion Degradation | 6.77 | | 5.87 |  |  |
| Mevalonate Pathway I | 6.12 | | 10.3 |  |  |
| Ketogenesis | 6.08 | | 4.95 |  |  |
| Estrogen Biosynthesis | 6.95 | | 5.9 |  |  |
| **RNA-Seq and microarray DEGs impacted specific pathways** | | | | | |
| *Aryl Hydrocarbon Receptor Signaling* | *5.59* | | *Tryptophan Degradation III (Eukaryotic)* | | *5.65* |
| *Zymosterol Biosynthesis* | *4.68* | | *PXR/RXR Activation* | | *3.02* |
| *iNOS Signaling* | *3.15* | | *Stearate Biosynthesis I (Animals)* | | *3.28* |
| *Aryl Hydrocarbon Receptor Signaling* | *5.59* | |  |  |  |
| *Hepatic Cholestasis* | *3.35* | |  |  |  |
| *FXR/RXR Activation* | *4.68* | |  |  |  |
| *NRF2-mediated Oxidative Stress Response* | *3.05* | |  |  |  |
| *Ethanol degradation* | *3.23* | |  |  |  |
| *Antioxidant Action of Vitamin C* | *3.62* | |  |  |  |

**Supplemental Table S3B:**  APAP impacted canonical pathways. The second column is computed –log (p-value) for RNA-Seq and the third column is for microarray. The RNA-Seq and microarray specific pathways are shown in italics

|  | | **-log_10_(p-value) RNA-Seq** | **-log_10_(p-value) Microarray** |  |
| --- | --- | --- | --- | --- |
| **RNA-Seq and microarray DEGs impacted common pathways** | | | |  |
| NRF2-mediated Oxidative Stress Response | 10.7 | | 7.67 |  |
| Glutathione-mediated Detoxification | 9.63 | | 6.35 |  |
| LPS/IL-1 Mediated Inhibition of RXR Function | 5.73 | | 6.31 |  |
| Glutathione Redox Reactions I | 4.86 | | 5.48 |  |
| Aryl Hydrocarbon Receptor Signaling | 4.05 | | 4.91 |  |
| **RNA-Seq and microarray DEGs impacted specific pathways** | | | | |
| *Thioredoxin Pathway* | 3.51 | | *Superpathway of Cholesterol Biosynthesis* | *4.61* |
| *Antioxidant Action of Vitamin C* | *3.16* | | *Superpathway of Serine and Glycine Biosynthesis I* | *3.05* |
| *Glycogen Biosynthesis II (from UDP-D-Glucose)* | *2.43* | |  |  |
| *Pyrimidine Ribonucleotides De Novo Biosynthesis* | *2.48* | |  |  |

**Supplemental Table S3C:**  MDA impacted canonical pathways. The second column is computed –log (p-value) for RNA-Seq and the third column is for microarray. The RNA-Seq and microarray specific pathways are shown in italics

|  | | **-log_10_(p-value) RNA-Seq** | **-log_10_(p-value) Microarray** |  |
| --- | --- | --- | --- | --- |
| **RNA-Seq and microarray DEGs impacted common pathways** | | | |  |
| Leukocyte Extravasation Signaling | 14.6 | | 5.9 |  |
| Fcγ Receptor-mediated Phagocytosis in Macrophages and Monocytes | 10.2 | | 5.1 |  |
| Agranulocyte Adhesion and Diapedesis | 8.1 | | 5.3 |  |
| PPARα/RXRα Activation | 5.8 | | 3.1 |  |
| Hepatic Cholestasis | 5.4 | | 3.5 |  |
| LXR/RXR Activation | 6.3 | | 6.8 |  |
| Hepatic Fibrosis / Hepatic Stellate Cell Activation | 4.5 | | 7.1 |  |
| Aryl Hydrocarbon Receptor Signaling | 3.8 | | 3.3 |  |
| Production of Nitric Oxide and Reactive Oxygen Species in Macrophages | 6.61 | | 3.6 |  |
| **RNA-Seq and microarray DEGs impacted specific pathways** | | | | |
| *Integrin Signaling* | 5.9 | | *Ethanol Degradation II* | *3.4* |
| *Sertoli Cell-Sertoli Cell Junction Signaling* | *4.4* | | *FXR/RXR Activation* | *4.9* |
| *Dendritic Cell Maturation* | *4.2* | |  |  |
| *RAR Activation* | *4.1* | |  |  |
| *RhoGDI Signaling* | *5.3* | |  |  |

**Supplemental Table S3D:**  CCl_4_ impacted canonical pathways. The second column is computed –log (p-value) for RNA-Seq and the third column is for microarray. The RNA-Seq and microarray specific pathways are shown in italics

|  | | **-log_10_(p-value)** | **-log_10_(p-value)** |  |  |
| --- | --- | --- | --- | --- | --- |
| **RNA-Seq and microarray DEGs impacted common pathways** | | | |  |  |
| EIF2 Signaling | 28 | | 20.9 |  |  |
| LPS/IL-1 Mediated Inhibition of RXR Function | 10.4 | | 12.5 |  |  |
| Regulation of eIF4 and p70S6K Signaling | 7.5 | | 10.6 |  |  |
| Unfolded protein response | 7.4 | | 10.6 |  |  |
| FXR/RXR Activation | 6.6 | | 10.6 |  |  |
| mTOR Signaling | 6.6 | | 5.82 |  |  |
| LXR/RXR Activation | 5.4 | | 10.3 |  |  |
| Superpathway of Cholesterol Biosynthesis | 4.9 | | 4.95 |  |  |
| PXR/RXR Activation | 3.8 | | 5.9 |  |  |
|  |  | |  |  |  |
| **RNA-Seq and microarray DEGs impacted specific pathways** | | | | | |
| *NRF2-mediated Oxidative Stress Response* | *5.6* | | *Bile Acid Biosynthesis, Neutral Pathway* | | *3.4* |
| *Estrogen Biosynthesis* | *3.2* | | *Ketogenesis* | | *3.2* |
| *Acetone Degradation I (to Methylglyoxal)* | *3.7* | | *Aryl Hydrocarbon Receptor Signaling* | | *3.2* |
| *Histidine Degradation III* | *3.5* | |  |  |  |
| *TR/RXR Activation* | *3.2* | |  |  |  |
| *Xenobiotic Metabolism Signaling* | *3.3* | |  |  |  |
| *Arginine Biosynthesis IV* | *3.2* | |  |  |  |
| *Endoplasmic Reticulum Stress Pathway* | *4.7* | |  |  |  |
