# Supplementary material for: Comparison of RNA-Seq and Microarray Gene Expression Platforms for the Toxicogenomic Evaluation of Liver From Short-Term Rat Toxicity Studies
Source: Front Genet. 2019 Jan 22;9:636. doi: 10.3389/fgene.2018.00636 (PMC6349826; doi:10.3389/fgene.2018.00636)
Supplement: TABLE S1 — Summary of RNA-Seq alignment statistics. [file Data_Sheet_3.zip › Supplemental_TableS4A_C.docx]

**Supplemental Table S4 A-C:** Upstream regulator analysis using Ingenuity Pathway Analysis. Top upstream regulators predicted based on the genes that were significantly different for A) ANIT, B) MDA and C) CCl_4_ and MDA treatments in RNA-Seq and microarray platforms are shown in column 1. The computed activation score and p-value for microarray and RNA-Seq are given in columns 2-3 and 4-6, respectively.

**Table S4 A**

| Upstream Regulator | Predicted Activation State | Activation z-score  (Microarray) | p-value  (Microarray) | Activation z-score  (RNA-Seq) | p-value  (RNA-Seq) |
| --- | --- | --- | --- | --- | --- |
| POR | Inhibited | -4.553 | 2.09E-27 | -4.563 | 1.94E-24 |
| PPARA | Inhibited | -4.363 | 1.51E-32 | -4.199 | 4.5E-32 |
| OGA | Inhibited | -3.606 | 2.23E-06 | -3.888 | 2.81E-05 |
| SMARCB1 | Inhibited | -3.466 | 3.98E-07 | -2.771 | 8.58E-16 |
| let-7 | Inhibited | -3.365 | 0.000175 | -5.358 | 1.94E-07 |
| mir-21 | Inhibited | -3.242 | 5.74E-06 | -3.78 | 1.38E-09 |
| miR-29b-3p | Inhibited | -3.233 | 0.00204 | -2.544 | 0.00339 |
| AGT | Activated | 4.414 | 9.53E-08 | 4.752 | 1.07E-08 |
| SREBF2 | Activated | 4.467 | 6.83E-20 | 4.953 | 2.74E-16 |
| CSF2 | Activated | 5.299 | 1.28E-11 | 6.298 | 9.94E-14 |

**Table S4 B**

| Upstream Regulator | Predicted Activation State | Activation z-score  (Microarray) | p-value  (Microarray) | Activation z-score  (RNA-Seq) | p-value  (RNA-Seq) |
| --- | --- | --- | --- | --- | --- |
| PPARA | Inhibited | -5.229 | 1.37E-26 | -4.432 | 8.36E-18 |
| Alpha catenin | Inhibited | -3.692 | 1.32E-07 | -4.442 | 7.12E-05 |
| HNF4A | Inhibited | -3.58 | 0.000327 | -3.261 | 0.00155 |
| PRDM1 | Inhibited | -3.353 | 0.00614 | -3.456 | 3.03E-05 |
| PPARGC1A | Inhibited | -3.235 | 6.39E-07 | -3.207 | 4.94E-05 |
| STAT1 | Activated | 5.091 | 3.45E-11 | 6.581 | 1.76E-09 |
| Interferon alpha | Activated | 5.482 | 1.46E-10 | 6.147 | 3.07E-08 |
| TNF | Activated | 5.66 | 6.04E-28 | 8.381 | 1.47E-36 |
| F2 | Activated | 5.688 | 1.11E-10 | 6.895 | 9.41E-11 |
| IFNG | Activated | 6.077 | 2.45E-24 | 9.496 | 1.11E-31 |

**Table S4 C**

| Upstream Regulator | Predicted Activation State | Activation z-score  (Microarray) | p-value  (Microarray) | Activation z-score  (RNA-Seq) | p-value  (RNA-Seq |
| --- | --- | --- | --- | --- | --- |
| RICTOR | Inhibited | -4.357 | 4.33E-06 | -5.546 | 5.81E-06 |
| INSIG1 | Inhibited | -3.504 | 5.5E-08 | -3.649 | 0.000193 |
| POR | Inhibited | -3.209 | 2.09E-10 | -3.488 | 3.03E-10 |
| HNF4A | Inhibited | -3.977 | 4.49E-10 | -3.386 | 1.06E-09 |
| ACOX1 | Inhibited | -4.239 | 3.48E-08 | -3.219 | 2.84E-10 |
| TCR | Activated | 3.655 | 0.0131 | 4.322 | 0.000104 |
| ERN1 | Activated | 3.228 | 4.23E-12 | 4.475 | 5.41E-17 |
| ATF4 | Activated | 4.231 | 7.48E-13 | 4.745 | 2.89E-14 |
| MYCN | Activated | 4.84 | 6.61E-16 | 4.777 | 1.4E-22 |
| XBP1 | Activated | 5.64 | 7.3E-25 | 7.219 | 7.21E-31 |
