# Supplementary material for: Comparison of RNA-Seq and Microarray Gene Expression Platforms for the Toxicogenomic Evaluation of Liver From Short-Term Rat Toxicity Studies
Source: Front Genet. 2019 Jan 22;9:636. doi: 10.3389/fgene.2018.00636 (PMC6349826; doi:10.3389/fgene.2018.00636)
Supplement: TABLE S1 — Summary of RNA-Seq alignment statistics. [file Data_Sheet_3.zip › Supplementary_TableS2.docx]

Supplementary Table S2: Summary of serum chemistries

|  |  | **Na** | **K** | **Cl** | **CO2** | **Urea** | **Glu** | **Crea** | **Ca** | **Phos** | **TBil** | **TP** | **AlbG** | **Trig** | **Chol** | **AlkP** | **ALT-A** | **AST-A** | **GLDH** | **GLOB** |
| --- | --- | --- | --- | --- | --- | --- | --- | --- | --- | --- | --- | --- | --- | --- | --- | --- | --- | --- | --- | --- |
| **Treatment** |  | **mmol/L** | **mmol/L** | **mmol/L** | **mmol/L** | **mg/dL** | **mg/dL** | **mg/dL** | **mg/dL** | **mg/dL** | **mg/dL** | **g/dL** | **g/dL** | **mg/dL** | **mg/dL** | **U/L** | **U/L** | **U/L** | **U/L** | **g/dL** |
|  |  |  |  |  |  |  |  |  |  |  |  |  |  |  |  |  |  |  |  |  |
| Corn oil  (5 ml/kg) |  | 145 | 5.9 | 100 | 25 | 16 | 113 | 0.4 | 11.9 | 10.7 | 0.1 | 6.0 | 4.0 | 72 | 77 | 349 | 64 | 72 | 8 | 2.0 |
|  |  | 143 | 5.9 | 102 | 24 | 11 | 81 | 0.4 | 11.7 | 10.7 | 0.2 | 5.7 | 3.7 | 67 | 73 | 283 | 53 | 78 | 10 | 2.0 |
|  |  | 145 | 5.5 | 102 | 24 | 14 | 112 | 0.4 | 11.9 | 10.8 | 0.1 | 5.9 | 3.8 | 82 | 92 | 305 | 39 | 75 | 7 | 2.1 |
| **Mean  ± S.E.M.** |  | **144  ± 1** | **5.8  ± 0.1** | **101  ± 1** | **24  ± 0** | **14  ± 1** | **102  ± 11** | **0.4  ± 0.0** | **11.8  ± 0.1** | **10.7  ± 0.0** | **0.1  ± 0.0** | **5.9  ± 0.1** | **3.8  ± 0.1** | **74  ± 4** | **81  ± 6** | **312  ± 19** | **52  ± 7** | **75  ± 2** | **8  ± 1** | **2.0  ± 0.0** |
|  |  |  |  |  |  |  |  |  |  |  |  |  |  |  |  |  |  |  |  |  |
|  |  |  |  |  |  |  |  |  |  |  |  |  |  |  |  |  |  |  |  |  |
|  |  |  |  |  |  |  |  |  |  |  |  |  |  |  |  |  |  |  |  |  |
|  |  |  |  |  |  |  |  |  |  |  |  |  |  |  |  |  |  |  |  |  |
|  |  |  |  |  |  |  |  |  |  |  |  |  |  |  |  |  |  |  |  |  |
| Alpha-naphthyl isocyanate  (100 mg/kg) |  | 140 | 6.5 | 100 | 25 | 16 | 104 | 0.4 | 11.5 | 9.4 | 6.6 | 5.5 | 3.2 | 105 | 315 | 562 | 120 | 412 | 227 | 2.3 |
|  |  | 145 | 6.7 | 95 | 27 | 18 | 161 | 0.5 | 12.3 | 10.2 | 10.2 | 6.1 | 3.7 | 59 | 342 | 936 | 319 | 647 | 82 | 2.4 |
|  |  | 135 | 6.3 | 95 | 24 | 15 | 132 | 0.4 | 11.7 | 8.1 | 5.6 | 5.6 | 3.1 | 78 | 470 | 768 | 264 | 1158 | 886 | 2.5 |
| **Mean  ± S.E.M.** |  | **140  ± 3** | **6.5  ± 0.1** | **97  ± 2** | **25  ± 1** | **16  ± 1** | **132  ± 16** | **0.4  ± 0.0** | **11.8  ± 0.2** | **9.2  ± 0.6** | **7.5  ± 1.4** | **5.7  ± 0.2** | **3.3  ± 0.2** | **81  ± 13** | **376  ± 48** | **755  ± 108** | **234  ± 59** | **739  ± 220** | **398  ± 247** | **2.4  ± 0.1** |
|  |  |  |  |  |  |  |  |  |  |  |  |  |  |  |  |  |  |  |  |  |
| Carbon tetrachloride  (1582 mg/kg) |  | 143 | 7.9 | 105 | 23 | 17 | 130 | 0.5 | 11.4 | 12.1 | 0.1 | 5.4 | 3.5 | 11 | 12 | 738 | 204 | 395 | 131 | 1.9 |
|  |  | 143 | 7.4 | 102 | 23 | 21 | 117 | 0.5 | 12.1 | 12.7 | 0.3 | 5.4 | 3.5 | 26 | 11 | 730 | 394 | 506 | 235 | 1.9 |
|  |  | 142 | 8.0 | 104 | 23 | 22 | 127 | 0.5 | 12.2 | 11.4 | 0.2 | 6.1 | 3.9 | 13 | 39 | 640 | 462 | 735 | 166 | 2.2 |
| **Mean  ± S.E.M.** |  | **143  ± 0** | **7.8  ± 0.2** | **104  ± 1** | **23  ± 0** | **20  ± 2** | **125  ± 4** | **0.5  ± 0.0** | **11.9  ± 0.3** | **12.1  ± 0.4** | **0.2  ± 0.0** | **5.6  ± 0.2** | **3.6  ± 0.1** | **17  ± 5** | **21  ± 9** | **703  ± 31** | **353  ± 77** | **545  ± 100** | **177  ± 31** | **2.0  ± 0.1** |

Supplementary Table (continued).

|  |  | **Na** | **K** | **Cl** | **CO2** | **Urea** | **Glu** | **Crea** | **Ca** | **Phos** | **TBil** | **TP** | **AlbG** | **Trig** | **Chol** | **AlkP** | **ALT-A** | **AST-A** | **GLDH** | **GLOB** |
| --- | --- | --- | --- | --- | --- | --- | --- | --- | --- | --- | --- | --- | --- | --- | --- | --- | --- | --- | --- | --- |
| **Treatment** |  | **mmol/L** | **mmol/L** | **mmol/L** | **mmol/L** | **mg/dL** | **mg/dL** | **mg/dL** | **mg/dL** | **mg/dL** | **mg/dL** | **g/dL** | **g/dL** | **mg/dL** | **mg/dL** | **U/L** | **U/L** | **U/L** | **U/L** | **g/dL** |
|  |  |  |  |  |  |  |  |  |  |  |  |  |  |  |  |  |  |  |  |  |
| Water (10 ml/kg) |  | 144 | 5.4 | 103 | 28 | 15 | 106 | 0.5 | 10.6 | 9.4 | 0.1 | 5.7 | 2.9 | 49 | 85 | 130 | 52 | 110 | 27 | 2.8 |
|  |  | 144 | 7.2 | 104 | 26 | 16 | 166 | 0.5 | 11.4 | 10.5 | 0.1 | 6.4 | 3.2 | 38 | 65 | 149 | 52 | 109 | 18 | 3.2 |
| **Mean**  **± S.E.M.** |  | **144**  **± 0** | **6.3**  **± 0.9** | **104**  **± 1** | **27**  **± 1** | **16**  **± 1** | **136**  **± 30** | **0.5**  **± 0.0** | **11.0**  **± 0.4** | **10.0**  **± 0.6** | **0.1**  **± 0.0** | **6.1**  **± 0.4** | **3.1**  **± 0.2** | **44**  **± 6** | **75**  **± 10** | **140**  **± 10** | **52**  **± 0** | **110**  **± 1** | **23**  **± 5** | **3.0**  **± 0.2** |
|  |  |  |  |  |  |  |  |  |  |  |  |  |  |  |  |  |  |  |  |  |
| Corn oil (2 ml/kg) |  | 143 | 5.8 | 102 | 29 | 11 | 119 | 0.5 | 10.6 | 9.1 | 0.0 | 6.0 | 3.2 | 67 | 58 | 162 | 51 | 76 | 18 | 2.8 |
|  |  | 143 | 7.1 | 103 | 28 | 13 | 181 | 0.5 | 11.1 | 9.4 | 0.0 | 6.0 | 3.1 | 52 | 58 | 212 | 54 | 92 | 15 | 2.9 |
|  |  | 143 | 5.7 | 102 | 28 | 16 | 109 | 0.5 | 10.4 | 8.7 | 0.1 | 5.8 | 2.9 | 44 | 90 | 144 | 54 | 137 | 21 | 2.9 |
| **Mean**  **± S.E.M.** |  | **143**  **± 0** | **6.2**  **± 0.5** | **102**  **± 0** | **28**  **± 0** | **13**  **± 1** | **136**  **± 23** | **0.5**  **± 0.0** | **10.7**  **± 0.2** | **9.1**  **± 0.2** | **0.0**  **± 0.0** | **5.9**  **± 0.1** | **3.1**  **± 0.1** | **54**  **± 7** | **69**  **± 11** | **173**  **± 20** | **53**  **± 1** | **102**  **± 18** | **18**  **± 2** | **2.9**  **± 0.0** |
|  |  |  |  |  |  |  |  |  |  |  |  |  |  |  |  |  |  |  |  |  |
| Acetaminophen (1000 mg/kg) |  | 144 | 6.2 | 103 | 26 | 12 | 185 | 0.5 | 11.3 | 10.5 | 0.2 | 6.1 | 3.0 | 62 | 128 | 185 | 80 | 94 | 18 | 3.1 |
|  |  | 141 | 7.6 | 104 | 23 | 14 | 237 | 0.6 | 11.3 | 9.7 | 0.2 | 6.6 | 3.4 | 64 | 119 | 269 | 140 | 98 | 22 | 3.2 |
|  |  | 142 | 6.2 | 102 | 28 | 13 | 199 | 0.4 | 10.5 | 10.3 | 0.4 | 6.0 | 3.0 | 102 | 112 | 139 | 70 | 85 | 11 | 3.0 |
| **Mean**  **± S.E.M.** |  | **142**  **± 1** | **6.7**  **± 0.5** | **103**  **± 1** | **26**  **± 1** | **13**  **± 1** | **207**  **± 16** | **0.5**  **± 0.0** | **11.0**  **± 0.3** | **10.2**  **± 0.2** | **0.3**  **± 0.1** | **6.2**  **± 0.2** | **3.1**  **± 0.1** | **76**  **± 13** | **120**  **± 5** | **198**  **± 38** | **97**  **± 22** | **92**  **± 4** | **17**  **± 3** | **3.1**  **± 0.1** |
|  |  |  |  |  |  |  |  |  |  |  |  |  |  |  |  |  |  |  |  |  |
| Diclofenac (10 mg/kg) |  | 143 | 7.2 | 102 | 28 | 12 | 156 | 0.5 | 11.4 | 9.2 | 0.1 | 6.4 | 3.3 | 87 | 120 | 141 | 51 | 79 | 12 | 3.1 |
|  |  | 143 | 5.9 | 102 | 26 | 12 | 174 | 0.5 | 11.3 | 8.7 | 0.1 | 6.1 | 3.2 | 63 | 64 | 178 | 54 | 83 | 21 | 2.9 |
|  |  | 145 | 7.7 | 100 | 27 | 16 | 228 | 0.5 | 12.7 | 10.5 | 0.0 | 6.5 | 3.2 | 50 | 75 | 146 | 52 | 78 | 12 | 3.3 |
| **Mean**  **± S.E.M.** |  | **144**  **± 1** | **6.9**  **± 0.5** | **101**  **± 1** | **27**  **± 1** | **13**  **± 1** | **186**  **± 22** | **0.5**  **± 0.0** | **11.8**  **± 0.5** | **9.5**  **± 0.5** | **0.1**  **± 0.0** | **6.3**  **± 0.1** | **3.2**  **± 0.0** | **67**  **± 11** | **86**  **± 17** | **155**  **± 12** | **52**  **± 1** | **80**  **± 2** | **15**  **± 3** | **3.1**  **± 0.1** |
|  |  |  |  |  |  |  |  |  |  |  |  |  |  |  |  |  |  |  |  |  |
| Ethanol (6000 mg/kg) |  | 145 | 7.1 | 106 | 26 | 12 | 96 | 0.5 | 10.8 | 10.9 | 0.1 | 5.4 | 2.9 | 70 | 71 | 138 | 89 | 77 | 11 | 2.5 |
|  |  | 147 | 4.6 | 98 | 0 | 11 | 177 | 0.0 | 9.3 | 6.4 | 0.0 | 4.7 | 2.2 | 559 | 141 | 252 | 56 | 108 | 0 | 2.5 |
|  |  | 143 | 8.1 | 101 | 0 | 14 | 174 | 0.0 | 10.0 | 10.3 | 0.0 | 5.3 | 2.7 | 98 | 139 | 182 | 92 | 266 | 0 | 2.6 |
| **Mean**  **± S.E.M.** |  | **145**  **± 1** | **6.6**  **± 1.0** | **102**  **± 2** | **9**  **± 9** | **12**  **± 1** | **149**  **± 27** | **0.2**  **± 0.2** | **10.0**  **± 0.4** | **9.2**  **± 1.4** | **0.0**  **± 0.0** | **5.1**  **± 0.2** | **2.6**  **± 0.2** | **242**  **± 159** | **117**  **± 23** | **191**  **± 33** | **79**  **± 12** | **150**  **± 59** | **4**  **± 4** | **2.5**  **± 0.0** |

Supplementary Table (continued).

|  |  | **Na** | **K** | **Cl** | **CO2** | **Urea** | **Glu** | **Crea** | **Ca** | **Phos** | **TBil** | **TP** | **AlbG** | **Trig** | **Chol** | **AlkP** | **ALT-A** | **AST-A** | **GLDH** | **GLOB** |
| --- | --- | --- | --- | --- | --- | --- | --- | --- | --- | --- | --- | --- | --- | --- | --- | --- | --- | --- | --- | --- |
| **Treatment** |  | **mmol/L** | **mmol/L** | **mmol/L** | **mmol/L** | **mg/dL** | **mg/dL** | **mg/dL** | **mg/dL** | **mg/dL** | **mg/dL** | **g/dL** | **g/dL** | **mg/dL** | **mg/dL** | **U/L** | **U/L** | **U/L** | **U/L** | **g/dL** |
|  |  |  |  |  |  |  |  |  |  |  |  |  |  |  |  |  |  |  |  |  |
| 35% Ethanol in water  (2 ml/kg) |  | 143 | 4.5 | 101 | 26 | 10 | 119 | 0.4 | 11.1 | 9.3 | 0.1 | 5.9 | 3.1 | 85 | 90 | 322 | 52 | 86 | 16 | 2.8 |
|  |  | 143 | 6.1 | 103 | 25 | 15 | 199 | 0.5 | 11.8 | 11.2 | 0.2 | 6.1 | 3.4 | 66 | 109 | 242 | 75 | 141 | 24 | 2.7 |
|  |  | 146 | 4.3 | 102 | 28 | 21 | 127 | 0.5 | 11.1 | 8.2 | 0.1 | 6.4 | 3.4 | 119 | 82 | 265 | 77 | 109 | 21 | 3.0 |
| **Mean**  **± S.E.M.** |  | **144**  **± 1** | **5.0**  **± 0.6** | **102**  **± 1** | **26**  **± 1** | **15**  **± 3** | **148**  **± 25** | **0.5**  **± 0.0** | **11.3**  **± 0.2** | **9.6**  **± 0.9** | **0.1**  **± 0.0** | **6.1**  **± 0.1** | **3.3**  **± 0.1** | **90**  **± 16** | **94**  **± 8** | **276**  **± 24** | **68**  **± 8** | **112**  **± 16** | **20**  **± 2** | **2.8**  **± 0.1** |
|  |  |  |  |  |  |  |  |  |  |  |  |  |  |  |  |  |  |  |  |  |
| Methylene dianiline  (25 mg/kg) |  | 143 | 6.7 | 101 | 28 | 13 | 215 | 0.5 | 11.9 | 10.8 | 0.2 | 5.6 | 3.2 | 35 | 77 | 282 | 53 | 68 | 17 | 2.4 |
|  |  | 141 | 7.4 | 102 | 25 | 12 | 196 | 0.5 | 11.8 | 11.2 | 0.1 | 6.1 | 3.4 | 63 | 159 | 270 | 67 | 80 | 18 | 2.7 |
|  |  | 143 | 5.9 | 103 | 24 | 13 | 221 | 0.4 | 12.1 | 10.1 |  | 6.0 | 3.3 | 104 | 108 | 239 | 52 | 70 | 18 | 2.7 |
| **Mean**  **± S.E.M.** |  | **142**  **± 1** | **6.7**  **± 0.4** | **102**  **± 1** | **26**  **± 1** | **13**  **± 0** | **211**  **± 8** | **0.5**  **± 0.0** | **11.9**  **± 0.1** | **10.7**  **± 0.3** | **0.1**  **± 0.0** | **5.9**  **± 0.2** | **3.3**  **± 0.1** | **67**  **± 20** | **115**  **± 24** | **264**  **± 13** | **57**  **± 5** | **73**  **± 4** | **18**  **± 0** | **2.6**  **± 0.1** |
|  |  |  |  |  |  |  |  |  |  |  |  |  |  |  |  |  |  |  |  |  |
| Methylene dianiline  (50 mg/kg) |  | 142 | 4.4 | 103 | 26 | 15 | 137 | 0.4 | 11.1 | 9.4 | 0.2 | 5.6 | 2.9 | 48 | 151 | 280 | 42 | 62 | 21 | 2.7 |
|  |  | 143 | 6.0 | 104 | 25 | 19 | 259 | 0.5 | 12.1 | 8.9 | 0.1 | 6.3 | 3.3 | 59 | 119 | 247 | 152 | 122 | 230 | 3.0 |
|  |  | 142 | 5.6 | 105 | 26 | 12 | 163 | 0.5 | 11.3 | 8.5 | 0.2 | 5.7 | 2.9 | 48 | 98 | 268 | 61 | 72 | 22 | 2.8 |
| **Mean**  **± S.E.M.** |  | **142**  **± 0** | **5.3**  **± 0.5** | **104**  **± 1** | **26**  **± 0** | **15**  **± 2** | **186**  **± 37** | **0.5**  **± 0.0** | **11.5**  **± 0.3** | **8.9**  **± 0.3** | **0.2**  **± 0.0** | **5.9**  **± 0.2** | **3.0**  **± 0.1** | **52**  **± 4** | **123**  **± 15** | **265**  **± 10** | **85**  **± 34** | **85**  **± 19** | **91**  **± 70** | **2.8**  **± 0.1** |
|  |  |  |  |  |  |  |  |  |  |  |  |  |  |  |  |  |  |  |  |  |
| Methylene dianiline  (100 mg/kg) |  | 143 | 6.1 | 103 | 23 | 13 | 189 | 0.5 | 12.7 | 9.1 | 0.6 | 5.6 | 2.5 | 40 | 125 | 402 | 208 | 284 | 100 | 3.1 |
|  |  | 142 | 5.7 | 102 | 27 | 17 | 175 | 0.5 | 11.9 | 6.9 | 2.2 | 5.8 | 2.8 | 83 | 222 | 728 | 222 | 503 | 62 | 3.0 |
|  |  | 143 | 5.7 | 104 | 26 | 16 | 167 | 0.5 | 12.0 | 9.2 | 0.8 | 5.5 | 2.6 | 59 | 172 | 419 | 150 | 312 | 101 | 2.9 |
| **Mean**  **± S.E.M.** |  | **143**  **± 0** | **5.8**  **± 0.1** | **103**  **± 1** | **25**  **± 1** | **15**  **± 1** | **177**  **± 6** | **0.5**  **± 0.0** | **12.2**  **± 0.3** | **8.4**  **± 0.8** | **1.2**  **± 0.5** | **5.6**  **± 0.1** | **2.6**  **± 0.1** | **61**  **± 12** | **173**  **± 28** | **516**  **± 106** | **193**  **± 22** | **366**  **± 69** | **88**  **± 13** | **3.0**  **± 0.1** |
