# Supplementary material for: Comparison of RNA-Seq and Microarray Gene Expression Platforms for the Toxicogenomic Evaluation of Liver From Short-Term Rat Toxicity Studies
Source: Front Genet. 2019 Jan 22;9:636. doi: 10.3389/fgene.2018.00636 (PMC6349826; doi:10.3389/fgene.2018.00636)
Supplement: TABLE S1 — Summary of RNA-Seq alignment statistics. [file Data_Sheet_3.zip › Supplemental_Table_S5A_B.docx]

**Table-S5 A: Pathways impacted by RNA-Seq Specific DEGs of ANIT and APAP. Columns 1 and 2 corresponds to ANIT and third and fourth for APAP**

| **Canonical Pathways** | **-log(p-value)** | **Canonical Pathways** | **-log(p-value)** |
| --- | --- | --- | --- |
| LPS/IL-1 Mediated Inhibition of RXR Function | 7.63 | NRF2-mediated Oxidative Stress Response | 4.03 |
| Aryl Hydrocarbon Receptor Signaling | 7.27 | Glutathione-mediated Detoxification | 3.82 |
| Cell Cycle Control of Chromosomal Replication | 5.83 | Vitamin-C Transport | 3.79 |
| Bupropion Degradation | 5.73 | Thioredoxin Pathway | 3.75 |
| Acetone Degradation I (to Methylglyoxal) | 5.54 | tRNA Charging | 3.11 |
| Estrogen Biosynthesis | 5.35 | Pyrimidine Ribonucleotides De Novo Biosynthesis | 2.84 |
| PPARα/RXRα Activation | 5.33 | Heme Degradation | 2.59 |
| Acute Phase Response Signaling | 5.14 | Antioxidant Action of Vitamin C | 2.41 |
| Sertoli Cell-Sertoli Cell Junction Signaling | 5.06 | Proline Biosynthesis II (from Arginine) | 2.37 |
| RhoA Signaling | 4.79 | Myo-inositol Biosynthesis | 2.37 |
| Antioxidant Action of Vitamin C | 4.59 | GDP-glucose Biosynthesis | 2.37 |
| IL-1 Signaling | 4.41 | Inhibition of Matrix Metalloproteases | 2.26 |
| RAR Activation | 4.39 | Glucose and Glucose-1-phosphate Degradation | 2.2 |
| Axonal Guidance Signaling | 4.25 | Bladder Cancer Signaling | 2.19 |
| FXR/RXR Activation | 4.22 | Xenobiotic Metabolism Signaling | 2.15 |
| Xenobiotic Metabolism Signaling | 4.07 | Pyrimidine Ribonucleotides Interconversion | 2.09 |
| Role of BRCA1 in DNA Damage Response | 3.91 | Histidine Degradation III | 2.06 |
| NRF2-mediated Oxidative Stress Response | 3.89 | Glutathione Redox Reactions I | 1.97 |
| Role of CHK Proteins in Cell Cycle Checkpoint Control | 3.88 |  |  |

**Table-S5 B: Pathways impacted by RNA-Seq Specific DEGs of CCl_4_ and MDA. Columns 1 and 2 corresponds to CCl_4_ and third and fourth for MDA**

| **Canonical Pathways** | **-log(p-value)** | **Canonical Pathways** | **-log(p-value)** |
| --- | --- | --- | --- |
| EIF2 Signaling | 16.8 | Leukocyte Extravasation Signaling | 7.95 |
| Regulation of eIF4 and p70S6K Signaling | 7.12 | CD28 Signaling in T Helper Cells | 5.78 |
| mTOR Signaling | 6.77 | Epithelial Adherens Junction Signaling | 4.9 |
| LPS/IL-1 Mediated Inhibition of RXR Function | 5.37 | PPARα/RXRα Activation | 4.49 |
| Bupropion Degradation | 4.78 | Germ Cell-Sertoli Cell Junction Signaling | 4.28 |
| NRF2-mediated Oxidative Stress Response | 4.47 | p70S6K Signaling | 4.12 |
| Acetone Degradation I (to Methylglyoxal) | 3.86 | Fcγ Receptor-mediated Phagocytosis in Macrophages and Monocytes | 4.12 |
| Germ Cell-Sertoli Cell Junction Signaling | 3.84 | Integrin Signaling | 4.1 |
| FXR/RXR Activation | 3.73 | Th2 Pathway | 4.07 |
| Estrogen Biosynthesis | 3.65 | Axonal Guidance Signaling | 3.92 |
| UDP-N-acetyl-D-glucosamine Biosynthesis II | 3.61 | IL-8 Signaling | 3.88 |
| Xenobiotic Metabolism Signaling | 3.42 | Tec Kinase Signaling | 3.87 |
| Folate Transformations I | 3.37 | Neuroinflammation Signaling Pathway | 3.8 |
| LXR/RXR Activation | 3.37 | Phagosome Formation | 3.76 |
| Superpathway of Melatonin Degradation | 3.17 | Hepatic Cholestasis | 3.69 |
| 14-3-3-mediated Signaling | 2.92 | 14-3-3-mediated Signaling | 3.63 |
| Melatonin Degradation I | 2.86 | Sertoli Cell-Sertoli Cell Junction Signaling | 3.54 |
| Histidine Degradation III | 2.82 | RAR Activation | 3.52 |
